# Identification and validation of m^6^A RNA methylation regulators with clinical prognostic value in Papillary thyroid cancer

**DOI:** 10.1186/s12935-020-01283-y

**Published:** 2020-05-29

**Authors:** Xinyi Wang, Xiaorui Fu, Junjia Zhang, Chengfeng Xiong, Shuyong Zhang, Yunxia Lv

**Affiliations:** 1 Queen Mary College, Medical Department, Nanchang University, Nanchang, Jiangxi People’s Republic of China; 2 Department of Breast and Endocrine Surgical Oncology, Graduate School of Medicine, Tohoku University, Sendai, Miyagi 980-8574 Japan; 3 Department of Thyroid Surgery, The Second Affiliated Hospital of Nanchang University, Nanchang, Jiangxi People’s Republic of China

**Keywords:** Papillary thyroid cancer, m^6^A, RNA methylation, TCGA, IGF2BP2

## Abstract

**Background:**

Papillary thyroid cancer (PTC) is a type of malignant tumor with excellent prognosis, accounting for more than 80% of thyroid cancer. Recently, numerous studies illustrated the importance of *N*^6^-methyladenosine (m^6^A) RNA modification to tumorigenesis, but it has never been reported in PTC.

**Methods:**

We downloaded data from The Cancer Genome Atlas (TCGA) and analyzed RNA expression, single nucleotide polymorphisms (SNPs) and copy number variations (CNVs) of 19 m^6^A RNA methylation regulators in PTC. Then we used nonnegative matrix factorization (NMF) to cluster patients into two m^6^A subtypes and compared them in overall survival (OS) and disease-free survival (DFS). The Weighted correlation network analysis (WGCNA) and univariate Cox proportional hazard model (CoxPH) were used to select genes for the construction of a m^6^A-related signature. The accuracy and prognostic value of this signature were validated by using receiver operating characteristic (ROC) curves, K-M (Kaplan–Meier) survival analysis, univariant and multivariant analyses.

**Results:**

CNVs and differential expression of m^6^A regulators were observed in PTC patients. Especially IGF2BP2 (Insulin-like growth factor 2 mRNA binding protein 2), which was most significantly overexpressed in tumor tissue. We chose 4 genes in the m^6^A-related module from WGCNA: IGF2BP2, STT3A, MTHFD1 and GSTM4, and used them to construct a m^6^A-related signature. The prognostic value of this signature was validated, and risk scores provided by the signature was the independent prognostic factor for PTC. A nomogram was also provided for clinical usage.

**Conclusions:**

We performed a comprehensive evaluation of the m^6^A RNA modification landscape of PTC and explored its underlying mechanisms. Our m^6^A-related signature was of great significance in predicting the DFS of patients with PTC. And IGF2BP2 was a gene worthy for further analysis as its strong correlation with DFS and clinical phenotypes of PTC.

## Background

Thyroid cancer is one of malignant tumors whose incidence are rapidly increasing in the world for both men and women. It can be classified into several subtypes: PTC, follicular thyroid cancer (FTC) and medullary thyroid cancer (MTC) [1]. PTC is the most common type of thyroid cancer, accounting for more than 80% of all cases. Generally, prognosis of patients with PTC is excellent, with 5-year-survival rate over 97% [2]. The 10-year and 15-year survival rates of papillary microcarcinoma, PTC which is smaller than 1 cm, are even over 99% [3]. In previous studies, lymph node metastasis has been proved to increase the risk of local recurrence without influencing survival in PTC [4]. Wada et al. indicated that patients without lymph node metastasis has nearly no chance of recurrence, while the recurrence rate of patients with lymph node metastasis is over 16% [5]. As a result, it is of greater significance to explore prognostic factors for DFS than OS.

Generally, DNA and histone protein are considered to be essential participants of reversible epigenetic modification which can regulate gene expression in mammal cells [6]. In recent years, reversible RNA modification, especially methylation, has been demonstrated to be another important component of gene expression regulation. The m^6^A RNA methylation, which was discovered in the 1970s, was the first example of reversible RNA methylation and wildly distributed in long non-coding RNAs and polyadenylated mRNAs [7, 8]. m^6^A has been observed within introns, internal exons, 3ʹ untranslated regions (3ʹUTRs) and stop codons, suggesting its addition can be earlier or simultaneous with RNA splicing [9, 10]. There are 3 types of m^6^A RNA methylation regulators: methyltransferases (writers), RNA binding proteins (readers), and demethylases (erasers). Writers are composed of METTL3, METTL14, METTL16, RBM15, RBM15B, WTAP and KIAA1429. Readers are comprised of YTHDC1/2, YTHDF1/2/3, IGF2BP1/2/3, HNRNPA2B1 and HNRNPC. FTO and ALKBH5 serve as erasers which perform demethylation activity [11].

Numerous studies showed that m^6^A RNA methylation played a role in the occurrence and progression of multiple malignant tumors, including hepatocellular carcinoma, colorectal carcinoma, breast cancer, glioblastoma and clear cell renal cell carcinoma [12–15]. Yongsheng li et al. have concluded the characteristics of m^6^A RNA methylation across 33 types of cancer and predicted that the mechanism of m^6^A RNA modification might be related with the activation or depression of some oncogenic pathways such as PI3K-AKT-mTOR signaling, G2M checkpoint, KRAS and P53 pathways [16]. METTL3 and IGF2BP2 have been proved to be over-expressed in colorectal carcinoma and promote the progression of cancer [17]. The RNA transcripts of SOX2 were methylated by METTL3 and bonded with IGF2BP2, resulting in regulation of SOX2 degradation. Yunhao Chen et al. demonstrated that WTAP can lead to post-transcriptional suppression of its downstream effector, ETS proto-oncogene1 (ETS1), and further contribute to the proliferation of hepatocellular carcinoma [18]. However, there has been no research which specifically explored the landscape of m^6^A RNA methylation and its relationship with DFS in PTC. In addition to expression levels of m^6^A RNA methylation regulators, SNPs and CNVs may also have prognostic value for PTC.

In this study, we performed a comprehensive evaluation of the landscape of m^6^A RNA methylation in PTC and explored its underlying mechanisms. The expression level, CNVs, SNPs and correlated clinical phenotypes of m^6^A RNA methylation regulators were analyzed to confirm the significance of m^6^A modification in PTC. By applying NMF, we divided patients from the TCGA cohort into two clusters (cluster1 and cluster 2) according to the expression of 19 m^6^A RNA methylation regulators and validated their differences in OS and DFS. In order to screen out essential genes for constructing a m^6^A-related signature, we performed WGCNA, univariant and multivariant analyses. Finally, we validated the accuracy and explored the underlying mechanism of the m^6^A-related signature by a series of analyses to illustrate its prognostic value.

## Methods

### Data download

The transcriptome data, somatic mutation data and clinical information of PTC patients were obtained from the TCGA database via the GDC data portal (https://portal.gdc.cancer.gov/repository). We downloaded RNA-seq (level 3, HTSeq-FPKM data) of 493 PTC patients (493 primary tumor tissue and 58 solid normal tissue) with complete clinical information from the TCGA database. For SNP, we downloaded “Masked Somatic Mutation” subtype of somatic mutation data and used the VarScan software to process it. We used a R package called “maftools” [19] to analyze and visualize the Mutation Annotation Format (MAF) of somatic variants. For CNV, the loss and gain of copy-number have been identified using segmentation analysis and GISTIC2.0 algorithm. The microarray data of papillary thyroid cancer patients was downloaded from GSE58545 (normal = 18, PTC = 27) datasets in the Gene Expression Omnibus (GEO) database. Oncomine database (http://www.oncomine.org) was used to validated mRNA levels of m^6^A regulators in PTC. Human Protein Atlas (http://www.proteinatlas.org) was used to validate expression levels of m^6^A RNA methylation regulators by immunohistochemistry [20]. A list of antibodies which were used in IHC samples were provided in Additional file 1: Table S1. We used Genotype-tissue expression (GTEx) dataset to compare expression levels of m^6^A regulators among different tissues and genders.

### Non-negative matrix factorization consensus clustering

To investigate the relationship between the expression of m^6^A regulators and clinical phenotypes in PTC, we clustered PTC samples from TCGA into 2 different clusters (cluster 1 and cluster 2) using NMF. The purpose of NMF was to identify potential characteristics in gene expression profiles by resolving the original matrix into two non-negative matrices [21]. Deposition was repeatedly performed, and its result was aggregated to acquire consensus clustering of PTC samples. The most suitable number of subtypes was decided according to cophenetic, dispersion and silhouette coefficients. NMF was performed by a R package called “NMF” [22]. The number of clusters *k* was chosen as 2, and the number of runs was set at 200. We also used a R package called “survival” to compare the OS and DFS between cluster 1 and cluster 2.

### Construction of co-expression module networks

The WGCNA was performed to establish the gene co-expression network to find trait-related modules by the R package “WGCNA” [23]. All genes and samples were filtered by good genes or good samples test. Filtered genes were used to construct a scale-free network by calculating the connection strength between genes. Scale-free R^2^ ranging from 0 to 1 was used to determine a scale-free topology model. To minimize effects of noise and spurious associations, the adjacency matrix was transformed into Topological Overlap Matrix (TOM). And TOM-based dissimilarity was used to form modules by dynamic tree cut. Here, we set minimal module size as 50 and cut height as 0.25. We used the KOBAS database to exert Kyoto Encyclopedia of Genes (KEGG) pathway enrichment and gene ontology (GO) analysis of the m^6^A-related module in WGCNA. When the P value was less than 0.05, the enriched pathway was considered to be statistically significant.

### Construction of the m^6^A-related risk signature

The patients with PTC from TCGA were randomly divided into a training set (N = 241) and a testing set (N = 240) using a R package called “caret”. For the training set, the univariate CoxPH was used to identify genes whose expression levels were statistically correlated with DFS, among all genes in the m^6^A-related module (P < 0.05). The m^6^A-related risk signature gave patients in training and testing sets risk scores based on genes weighted value which was calculated by a linear combination of Cox coefficient and gene expression:

$${\text{Risk score}} = \mathop \sum \limits_{i = 0}^{N} \left( {\text{Expi * Coei}} \right).$$

And patients were classified into low-risk and high-risk group according to the median of risk scores.

### Validation of the m^6^A-related signature for colon cancer

The Chi square test was performed to confirm that there was no selection bias in classification of training and testing set. The univariate and multivariate analyses were conducted for both the m^6^A-related signature and clinical factors. The Kaplan–Meier (K–M) survival curves and log-rank test were generated to evaluate the difference in DFS between high‐risk group and low‐risk group in total TCGA PTC cohort, training set and testing set. We performed ROC curves to measure the prognostic capacity of our signature using a R package called “survivalROC”. A nomogram was used to predict cancer prognosis. In the TCGA datasets, all genes in the signature were included to generate the nomogram which can investigate the chancer of 1-, 3- and 5-year DFS of patients with PTC.

### Statistical analysis

We exerted Mann–Whitney U tests to compare the expression levels of m6A RNA methylation regulators in different subgroups (normal tissues/primary tumor tissue, cluster1/cluster2). Chi square test was performed to confirm the difference in CNVs between normal and tumor tissues. Chi square test was also used to analyze the difference in clinical phenotypes between cluster 1 and cluster 2, as well as high-risk group and low-risk group. The relationships between m^6^A regulators and other genes in the m^6^A-related module were analyzed by calculating the Spearman correlation coefficients.

## Results

### m^6^A RNA methylation regulators had different expression level in PTC and normal tissues

**Fig. 1:**
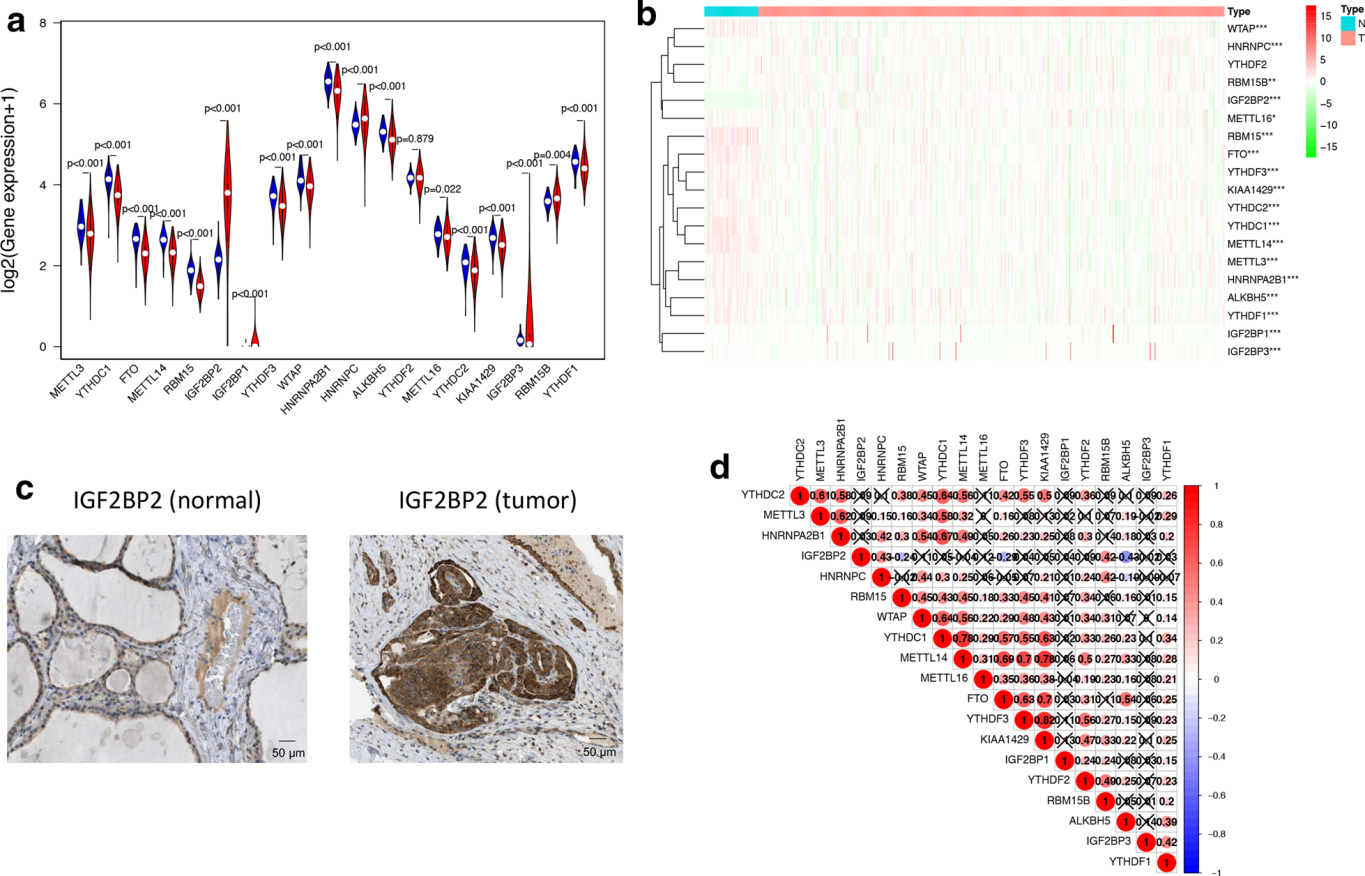
The overview of m^6^A RNA methylation regulators in PTC. **a** A Vioplot which visualized the differentially expressed m^6^A regulators in PTC (blue represents normal tissues and red represents tumor tissues). **b** The mRNA levels of 19 m^6^A regulators in PTC. Red means this gene is up-regulated while green means this gene is down-regulated (*P < 0.05, **P < 0.01, ***P < 0.001). **c** The validation of IGF2BP2 expression level by immunohistochemical staining. The result showed that the expression level of IGF2BP2 was higher in PTC tissue than normal thyroid tissue. **d** Spearman correlation analysis of the 19 m^6^A regulators in PTC. The “X” represents that the correlation between this pair of genes does not have statistical significance (P > 0.05). The number in every box is the Spearman correlation coefficient between two genes

Using transcriptome data from TCGA database, we analyzed the mRNA levels of 19 m^6^A regulators in PTC and para-tumor normal tissues (Fig. 1a, b, Additional file 2: Table S2). Except YTHDF2, 18 of 19 m^6^A regulators were differentially expressed in PTC and normal thyroid tissues. METTL3, YTHDC1, FTO, METTL14, RBM15, YTHDF3, WTAP, HNRNPA2B1, ALKBH5, METTL16, YTHDC2, KIAA1429, IGF2BP3, RBM15B and YTHDF1 had lower expression levels in tumor tissues, while IGF2BP2, IGF2BP1 and HNRNPC had higher expression level in tumor tissues. Among all of these genes, only IGF2BP2 had significantly higher expression pattern and distinguishable protein expression in tumor tissue compared to normal tissue (Fig. 1c). We also validated these results in the GEO database (Additional file 3: Table S3) and found 14 differentially expressed m^6^A RNA methylation regulators with 10 of them was the same as the analysis of TCGA. The expression of these genes was also further validated in the Oncomine database (Additional file 4: Figure S1) and Human Protein Atlas database (Fig. 1c and Additional file 5: Figure S2). Then we explored the relationships between 19 m^6^A RNA methylation regulators by Spearman correlation analysis (Fig. 1d). The relationships between each two of them were almost positively correlated, and YTHDF3 and KIAA1429 were most relevant (Cor = 0.82). However, there were also some genes which were negatively correlated, such as IGF2BP2 and ALKBH5 (Cor = − 0.43).

### CNVs and SNPs of m^6^A RNA methylation regulators can serve as prognostic factors for PTC

**Fig. 2:**
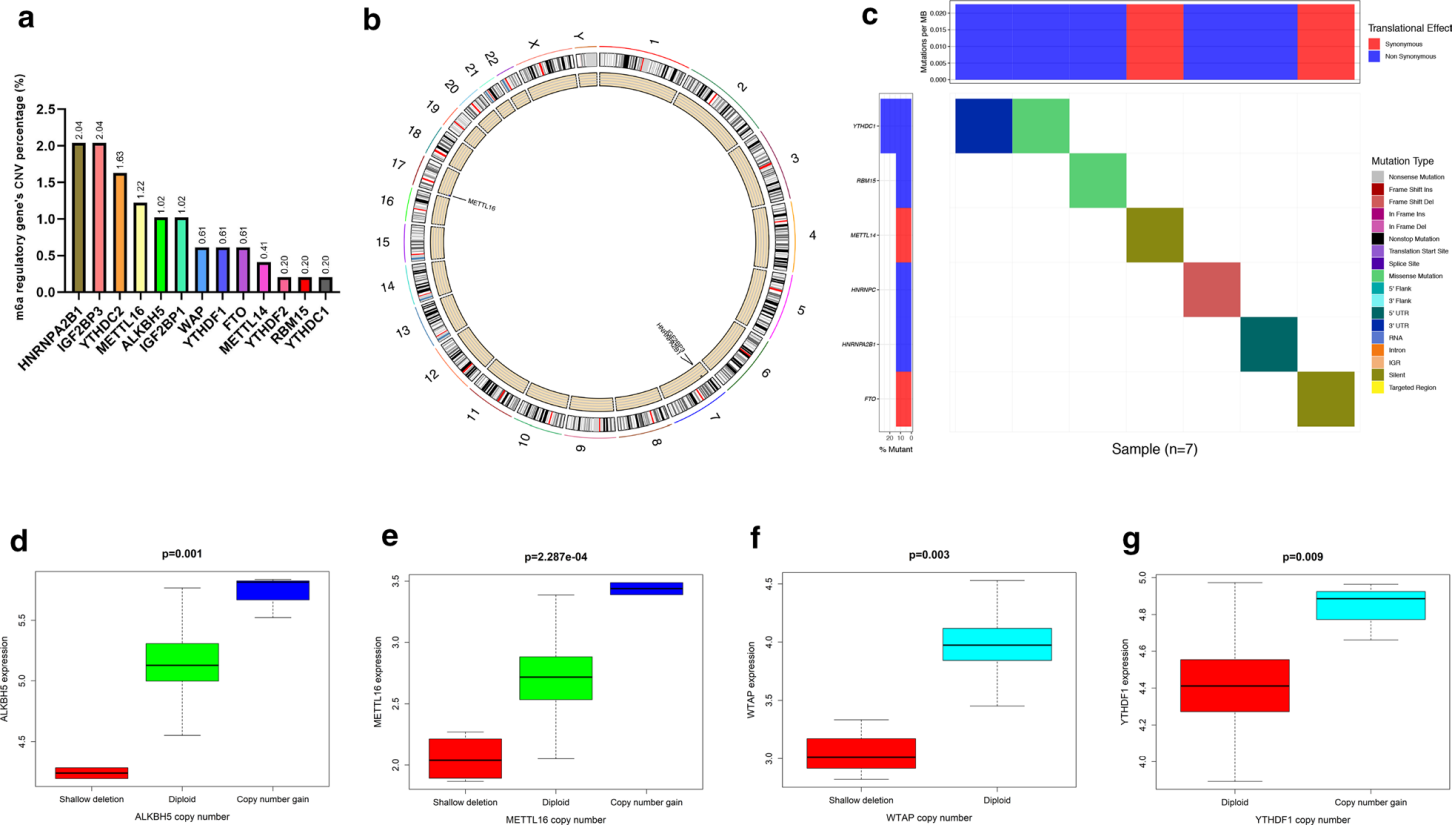
CNVs and SNPs analysis of m^6^A regulators. **a** The percentage of patients with CNVs in 13 m^6^A regulators. **b** The chromosome location of genes with frequently CNVs (HNRNPA2B1, IGF2BP3 and METTL16). **c** The SNPs of YTHDC, RBM15, METTL14, HNRNPC, HNRNPA2B1 and FTO were observed in 7 patients. **d**–**g** The copy numbers of ALKBH5, METTL16, WTAP and YTHDF1 were correlated with gene expression levels in PTC tissues

Among the 505 cases, CNVs of the 13 m6A RNA methylation regulators were frequently observed (Fig. 2a). In detail, two m6A “reader” genes HNRNPA2B1 and IGF2BP3 had the highest frequency of CNV events (2.04%, 2.04%) followed by YTHDC2 (1.63%) and METTl16 (1.22%). We performed Chi square test to analyze the difference in CNVs between normal tissues and PTC tissues, and found HNRNPA2B1 (P = 0.004235), IGF2BP3 (P = 0.004235) and METTL16 (P = 0.0040631) were of statistical significance (Additional file 6: Table S4). The chromosome position of HNRNPA2B1, IGF2BP3 and METTL16 were shown in Fig. 2b. Furthermore, we evaluated the correlation between the copy number and mRNA level of 19 m^6^A regulators, and found higher copy number of 4 genes were corresponded with higher expression level: ALKBH5 (P = 0.001), METTL16 (P = 2.287e−04), WTAP (P = 0.003) and YTHDF1 (P = 0.009) (Fig. 2d–g). SNPs of YTHDC1, RBM15, METTL14, HNRNPC, HNRNPA2B1 and FTO were found merely in 7 independent samples (Fig. 2c). CNVs and SNPs may influence the expression level and biological function of m6A RNA methylation regulators, and further effected the activities of RNA modification.

### Two m^6^A subgroups were different in clinical phenotypes and DFS

**Fig. 3:**
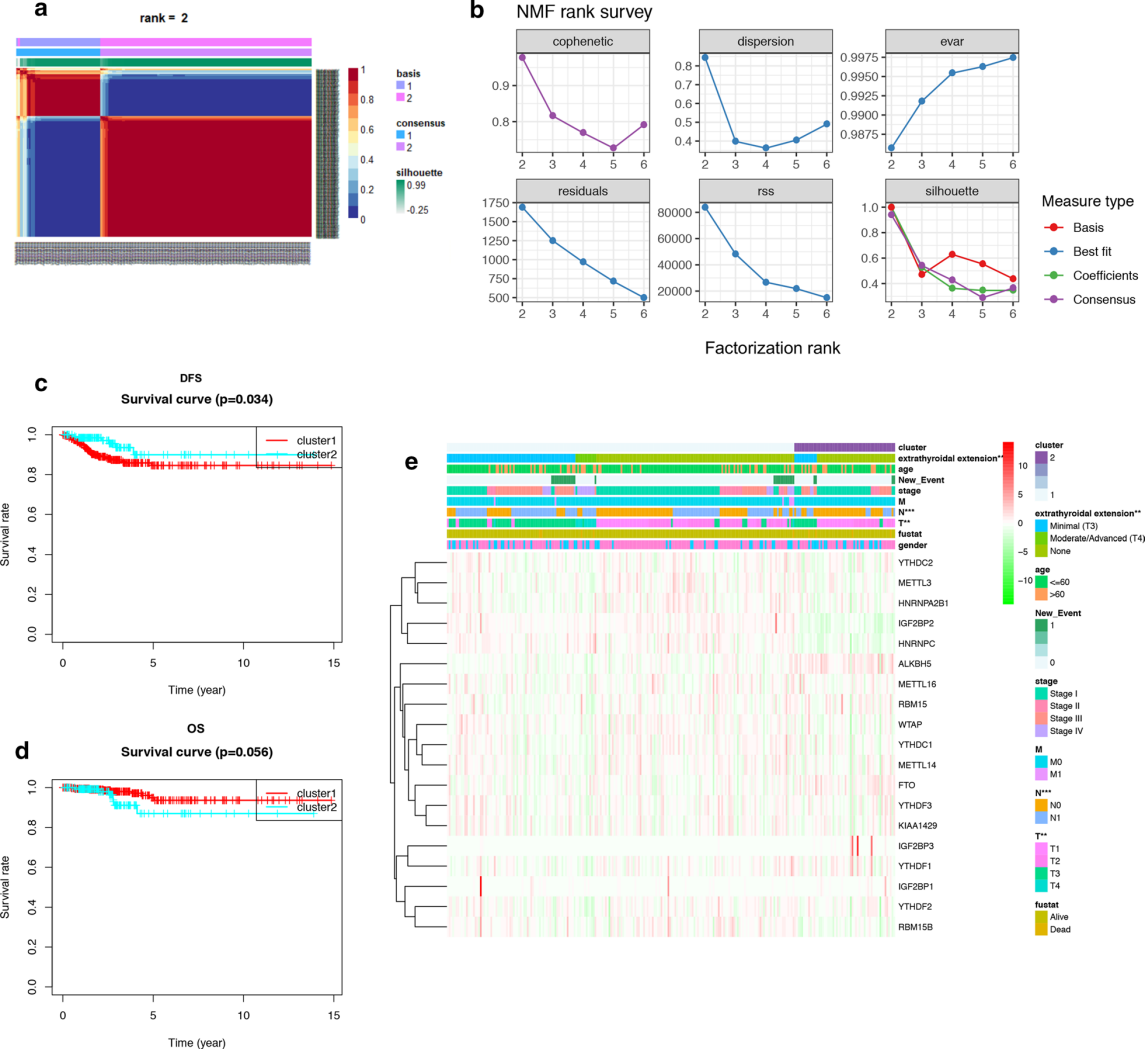
Identification of consensus clusters by m6A regulators. **a** The consensus map of NMF clustering results in the total TCGA cohort. Patients were divided into cluster 1 and cluster 2 according to the expression profiles of 19 m6A regulators. **b** The relationship between cophenetic, dispersion, evar, residuals, rss and silhouette coefficients with respect to number of clusters. **c**, **d** The survival curve of DFS (P = 0.034) and OS (P = 0.056) in cluster 1 and cluster 2. **e** The correlation analysis of m6A methylation regulators and clinical phenotypes in cluster 1 and cluster 2. The result showed that these 2 clusters has statistical differences in extrathyroidal extension, T and N classifications (*P < 0.05, **P < 0.01, ***P < 0.001)

The total TCGA cohort were clustered into 2 subgroups (cluster 1: n = 352 and cluster 2: n = 141) by applying NMF (Fig. 3a, b), according to expression levels of 19 m^6^A regulators in PTC samples. To better understand the clustering result and its relationships with survival outcomes and clinical phenotypes, we compared the OS and DFS between cluster 1 and cluster 2 and found cluster 2 had better DFS than cluster 1 (P = 0.034, Fig. 3c). But there was no statistical difference between the OS of cluster 1 and cluster 2 (P = 0.056, Fig. 3d). These results demonstrated that m^6^A RNA methylation may have strong correlation with DFS of PTC patients. As shown in the heatmap, IGF2BP1, WTAP, FTO, IGF2BP3 and ALKBH5 had higher expression level in cluster 2, while IGF2BP2, RBM15B, HNRNPC were significantly down-regulated in cluster 2 (Fig. 3e, Additional file 7: Table S5). We also found these 2 clusters were different in extrathyroidal extension (P < 0.01), T (P < 0.01) and N (P < 0.001) classifications, suggesting m^6^A RNA methylation may also related to clinical phenotypes and progression of PTC.

### Detection of DFS-related m^6^A regulator and its correlated module by WGANA

**Fig. 4:**
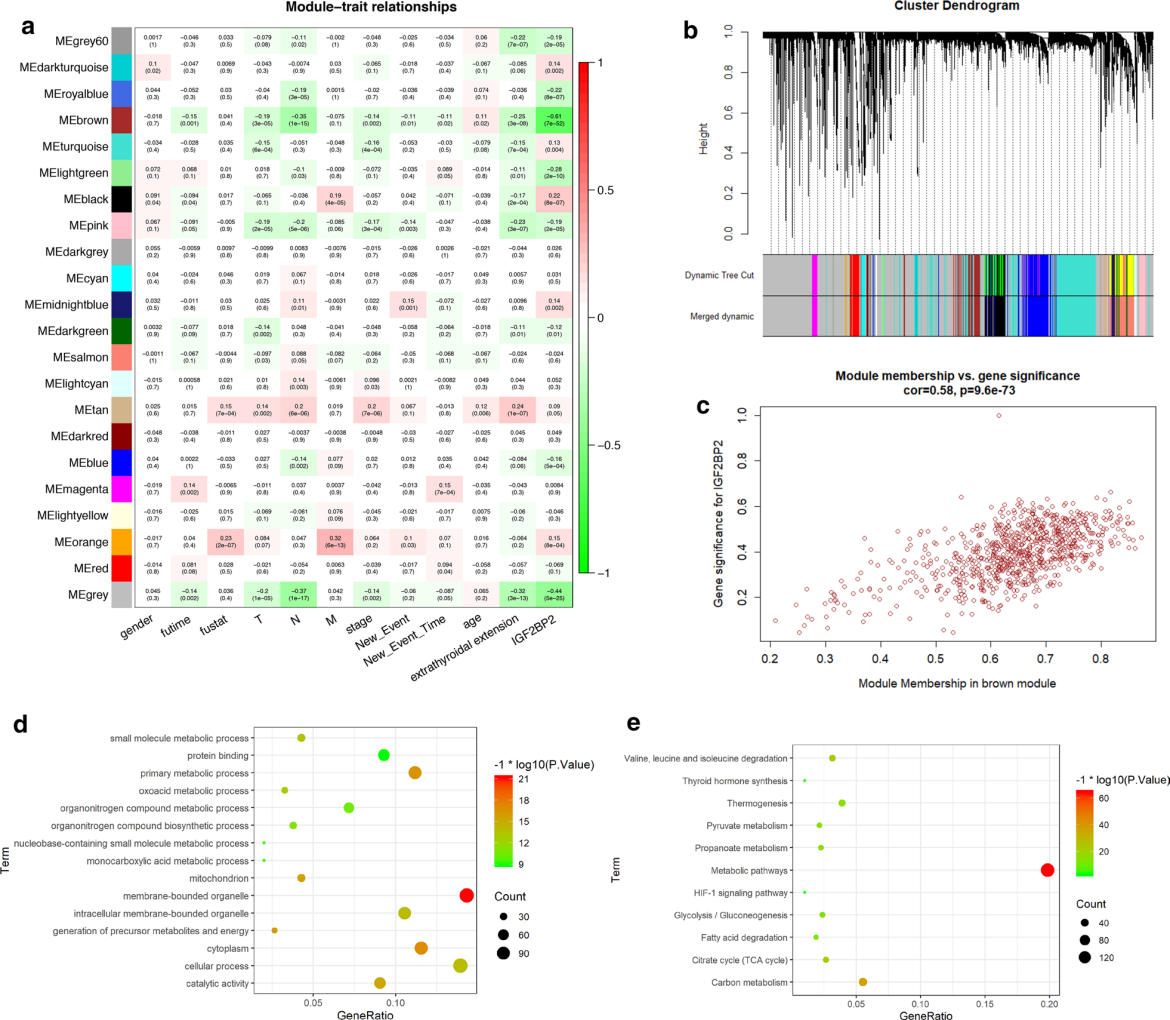
Detection and validation of m^6^A-related module by WGCNA. **a** Heatmap of the correlation between gene modules and the clinical phenotypes of PTC. The brown module was the most correlated module of IGF2BP2 expression level. **b** Hierarchical cluster analysis was performed to detect co-expression modules with corresponding colors. **c** The correlation analysis between membership (MM) in brown module and gene significance (GS) for IGF2BP2. **d**, **e** Bubble chart of GO and KEGG results of brown module

The univariant CoxPH was performed to identify m^6^A RNA methylation regulators which were prognostic factors for OS and DFS of patients with PTC (Table 1). We found that FTO (HR = 1.57, P = 0.044), RBM15 (HR = 3.84, P = 0.012), YTHDF3 (HR = 1.29, P = 0.009) and KIAA1429 (HR = 1.76, P = 0.042) were related with the overall survival rate, while only IGF2BP2 (P = 0.0006) was related with the DFS of PTC patients. As we mentioned before, the prognosis of PTC patient is excellent, and the recurrence of tumor is one of the biggest challenges at present. As a result, it is of greater value for us to study the mechanism of genes related to DFS of PTC patient. By WGCNA, we identified 22 co-expression modules and analyzed their association with 12 clinical phenotypes, including futime, fustat, TNM classification, stage, age, gender, new-event, new-event time, extrathyroidal extension and IGF2BP2 expression (High-expression and Low-expression) (Fig. 4a–c, Additional file 8: Figure S3). Except the grey module which contained non-clustering genes, the brown module was the most correlated module of IGF2BP2 expression (r = − 0.61, P = 7e−52). The brown module was also correlated with futime (r = − 0.15, P = 0.001), T (r = − 0.19, P = 3e−05), N (r = − 0.35, P = 1e−15), stage (r = − 0.14, P = 0.002), new-event (r = − 0.11, P = 0.01), new-event time (r = − 0.11, P = 0.02), age (r = 0.11, P = 0.02) and extrathyroidal extension (r = − 0.25, P = 3e−08). The result of KEGG and GO analyses showed that the brown module was related to membrane-bounded organelle, multiple metabolic process, thyroid hormone synthesis and HIF-1signaling pathway (Fig. 4d, e), especially metabolic pathways in the central carbon metabolism, such as pyruvate metabolism, citrate cycle, propanoate metabolism and glycolysis/gluconeogenesis.

**Table 1:** Univariant CoxPH analysis of OS and DFS

uniCox analysis								
Gene	Survival analysis				Disease free analysis			
	HR	HR.95L	HR.95H	P-value	HR	HR.95L	HR.95H	P-value
METTL3	0.973958	0.742174	1.278129	0.849081	0.981439	0.836601	1.151353	0.818114
YTHDC1	1.036531	0.852548	1.26022	0.718934	0.977604	0.873975	1.093522	0.691972
FTO	1.573325	1.012161	2.44561	*0.044044*	0.78099	0.577311	1.056529	0.108867
METTL14	1.25211	0.688421	2.277356	0.461329	0.977691	0.685448	1.394532	0.900902
RBM15	3.843867	1.338806	11.03619	*0.012343*	0.858734	0.388698	1.897167	0.70649
IGF2BP2	0.919768	0.8431	1.003408	0.059652	1.097637	1.040737	1.157648	*0.000603*
IGF2BP1	4.527263	0.510412	40.156	0.175085	0.183686	0.000123	274.5852	0.649576
YTHDF3	1.290726	1.065857	1.563037	*0.008975*	0.999697	0.891851	1.120584	0.995846
WTAP	0.994085	0.811996	1.217007	0.954173	0.916739	0.821487	1.023036	0.120403
HNRNPA2B1	0.991969	0.959783	1.025234	0.631828	0.983031	0.963915	1.002526	0.0876
HNRNPC	0.975073	0.928021	1.024511	0.317143	1.020512	0.987934	1.054164	0.219977
ALKBH5	1.04919	0.991575	1.110154	0.095643	0.957849	0.913425	1.004433	0.075499
YTHDF2	1.088042	0.895735	1.321636	0.395142	0.939033	0.833287	1.058199	0.302091
METTL16	1.021759	0.712861	1.464507	0.906707	1.001206	0.802832	1.248598	0.991461
YTHDC2	1.812616	0.943702	3.481584	0.074104	1.020589	0.686415	1.517452	0.919788
KIAA1429	1.756042	1.020936	3.020446	*0.041867*	1.065358	0.779387	1.456256	0.691363
IGF2BP3	1.097294	0.926038	1.30022	0.283528	0.155158	0.002606	9.23819	0.371512
RBM15B	1.024573	0.807027	1.300762	0.841987	1.077647	0.933178	1.244482	0.30856
YTHDF1	1.066432	0.990667	1.147991	0.087156	1.013381	0.948002	1.083268	0.696068

Italic values are statistically significant

### Construction and verification of the m^6^A-related risk signature

**Fig. 5:**
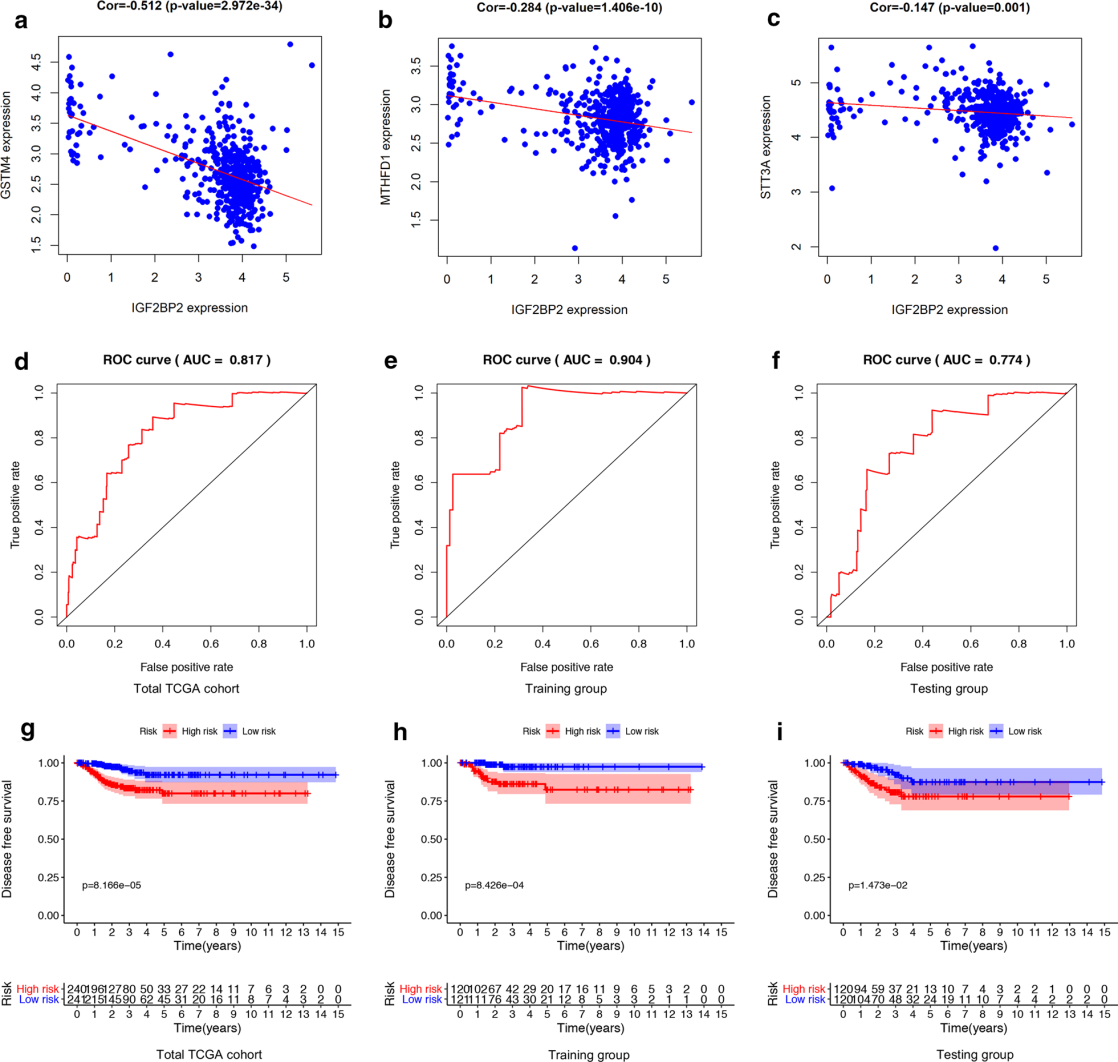
Construction and validation of m^6^A-related signature. **a**–**c** The Pearson correlation coefficients between IGF2BP2 and each gene in the signature. **d**–**f** The ROC curves of patients with PTC in the total TCGA cohort, training group and testing group. **g**–**i** Comparing DFS in high-risk and low-risk groups by performing K–M survival curves in the total TCGA cohort, training group and testing group

We randomly divided patients in the total TCGA cohort into training set and testing set. Then another univariant CoxPH was performed in the training set to filter genes which were related to DFS of PTC, among 796 genes in the brown module (Additional file 9: Table S6). We identified 4 genes: IGF2BP2 (HR = 1.19, P = 0.0002), STT3A (HR = 0.89, P = 0.033), MTHFD1 (HR = 1.32, P = 0.044) and GSTM4 (HR = 1.12, P = 0.049). The expression levels of STT3A, MTHFD1 and GSTM4 were strongly correlated with IGF2BP2 (Fig. 5a–c). These 4 genes were used to construct the m^6^A-related risk signature via multivariate CoxPH regression model (Additional file 10: Table S7). Risk scores of patients were calculated as follows:

$${\text{Risk score }} = \left( {0.390 \times {\text{MTHFD}}1} \right) + \left( {0.167 \times {\text{IGF}}2{\text{BP}}2} \right) + \left( {0.152 \times {\text{GSTM}}4} \right) + ( - 0.133 \times {\text{STT3A}})$$

**Fig. 6:**
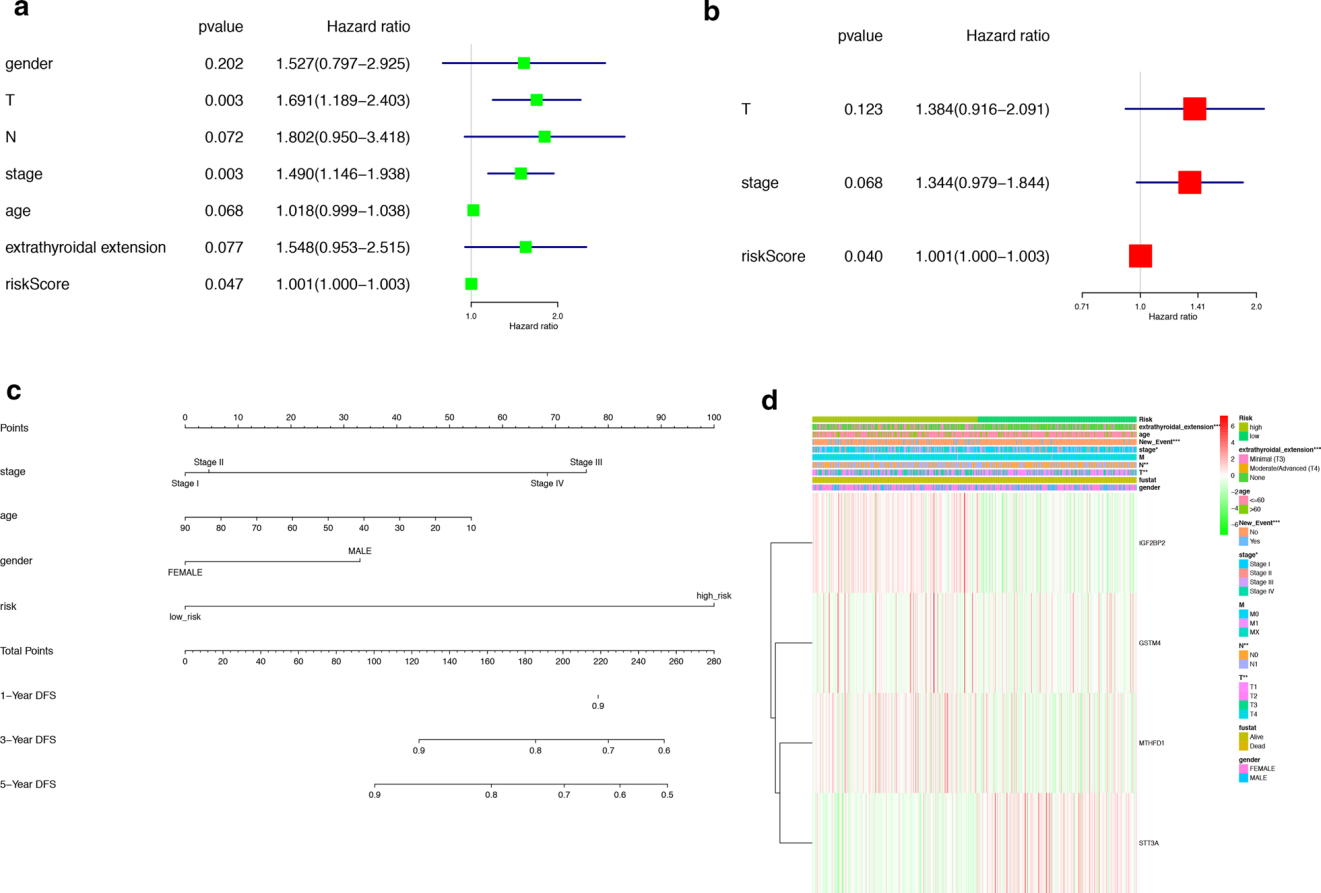
Analysis of prognostic factors for PTC. **a** Univariant CoxPH of risk score and 6 clinical phenotypes (gender, T, N, stage, age, extrathyroidal extension). **b** Multivariant CoxPH of T, stage and risk score. **c** A nomogram performed based on prognostic factors found in CoxPH. **d** A heatmap which showed the risk scores, clinical phenotypes and gene expression (IGF2BP2, GSTM2, MTHFD1 and STT3A) of patient with PTC. A Chi square test was also performed to evaluate the relationships among risk score and 9 clinical phenotypes (*P < 0.05, **P < 0.01, ***P < 0.001)

**Fig. 7:**
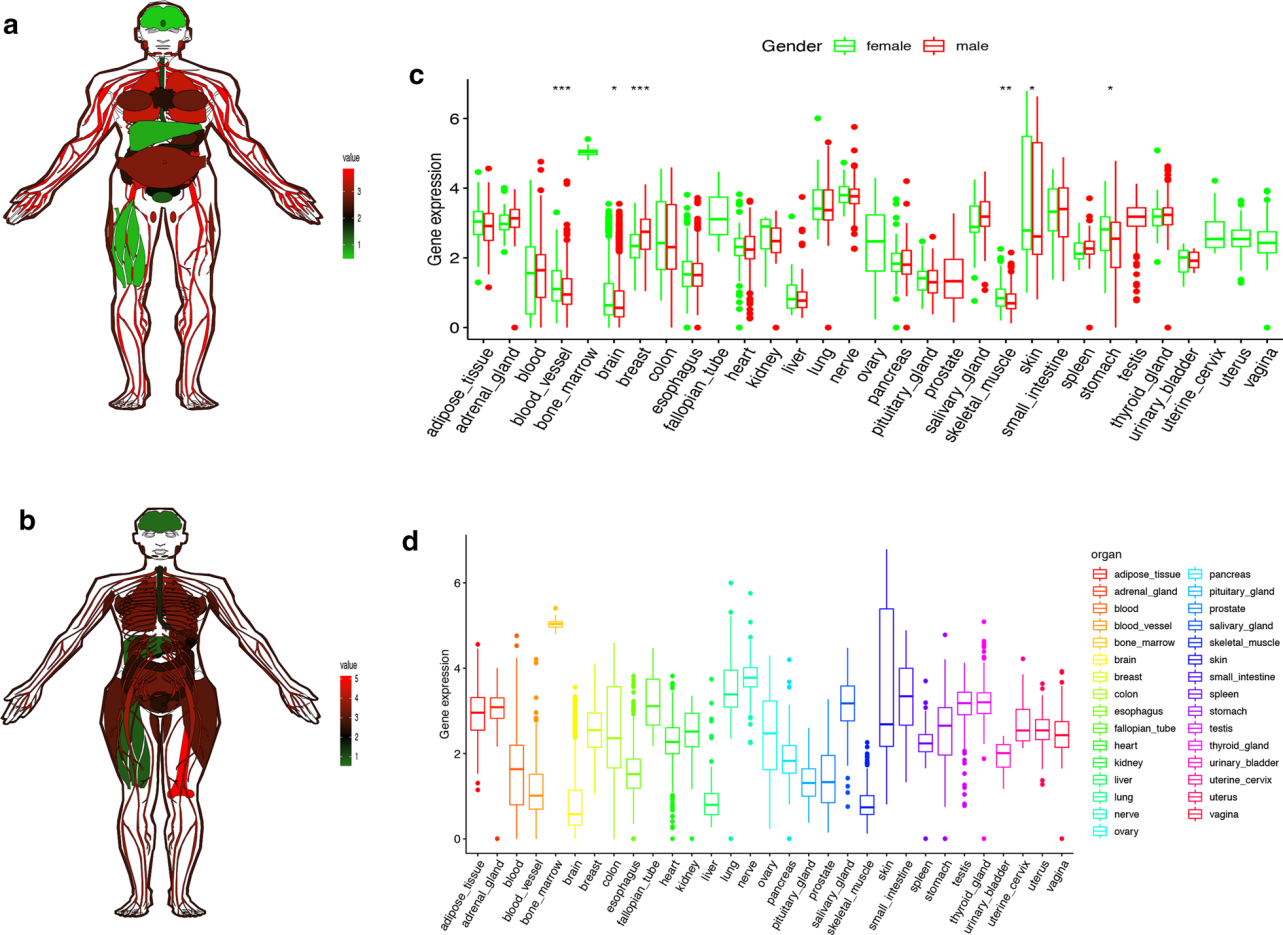
The expression landscape of IGF2BP2 in different genders and organs. **a**, **b** The expression levels of IGF2BP2 in different organs of male and female. Red represented high level of IGF2BP2 while green represented low level. **c** Comparing the expression of IGF2BP2 in 31 tissues of male and female by Mann–Whitney test (*P < 0.05, **P < 0.01, ***P < 0.001). **d** The relative expression level of IGF2BP2 in 31 types of human tissues

Patients were divided into high-risk and low-risk groups with the median risk score used as the cutoff value. The ROC curve analysis in total TCGA PTC cohort (AUC = 0.817, Fig. 5d), training set (AUC = 0.904, Fig. 5e) and testing set (AUC = 0.774, Fig. 5f), revealing promising prognosis value of the signature for PTC disease-free survival. The K–M survival curves were performed to illustrate the difference between the high-risk and low-risk groups in DFS: total TCGA PTC cohort (P = 8.166e−05, Fig. 5g), training set (P = 8.426e−04, Fig. 5h) and testing set (P = 1.473e−02, Fig. 5i). All of these analyses showed that patients in low-risk group had better prognosis than high-risk group and this m^6^A-related signature was of strong accuracy in predicting the DFS of patients with PTC. Univariant and multivariant CoxPH showed that T classification (HR = 1.691, P = 0.003), stage (HR = 1.49, P = 0.003) and risk score (HR = 1.001, P = 0.047) were prognostic factors for PTC (Fig. 6a), and only risk score was the independent prognostic factor (HR = 1.001, P = 0.04, Fig. 6b). Furthermore, a prognostic nomogram was constructed to predict DFS of individual patients with PTC (Fig. 6c). In Fig. 6d, we assessed whether there was statistical difference in clinical phenotypes between high-risk and low-risk groups by Chi square test. The heatmap indicated that high‐risk group was corresponded to advanced stage, higher level of T and N classifications, new tumor event and extrathyroidal extension in total TCGA PTC cohort. Finally, to better understand the expression of IGF2BP2 in human tissues, we used GTEx dataset to explore the landscape of IGF2BP2 in different genders and organs. The expression patterns of IGF2BP2 were similar in most organs of female and male, but were significantly different in blood vessel, brain, breast, skeletal muscle, skin and stomach (Fig. 7a–c). The tissue-specificity of IGF2BP2 is of great value to explore as it can provide clues for therapy and diagnosis. IGF2BP2 had high expression level in bone marrow and low expression level in brain, liver and skeletal muscle. These organs and malignant tumor originated from them can become potential objects of study (Fig. 7d).

## Discussion

Over past decades, the occurrence of PTC has been proved to be correlated with external radiation exposure, dietary iodine content and resultant disturbance of thyroid stimulating hormone (TSH) level [24]. Nowadays, increasing investigations begin to focus on acquired genetic changes that can distinguish PTC from para-tumor normal tissue, which has greatly expanded our knowledge of the molecular pathogenesis of PTC. Several biomarkers have been used in clinic, such as RET/PTC rearrangement, PAX8-PPARγ rearrangement, BRAF and RAS mutations [25–27]. As a promising field of cancer biology, m^6^A RNA modification has been verified to participant in developing several types of malignant tumor. However, there has been no research which explore the role and mechanism of m^6^A RNA modification in the progression of PTC. Here, we discovered some m^6^A RNA methylation regulators whose expression level, CNVs and SNPs were strongly correlated with PTC, including IGF2BP2, HNRNPA2B1 and IGF2BP3. They are all “readers” which can selectively bind to and change the secondary structures of m^6^A-containing RNAs. This process results in regulated degradation of targeted RNAs and can be reversibly tuned via m^6^A methylation and demethylation [28]. Readers may also affect RNA splicing, storage, trafficking and translation [29]. Overexpression of IGF2BP2 has been indicated to be related to poor survival of patients with colorectal cancer, acute myelocytic leukemia and metaplastic breast cancer [30, 31]. Recently, K.Wang et al. demonstrated that the progression of thyroid carcinoma can be promoted by METTL3 and IGF2BP2 through m6A methylation on TCF1 mRNA and activation of Wnt signaling pathway in thyroid cancer [32]. The relationship between IGF2BP2 and PI3K/Akt signaling pathway has been discussed, suggesting up-regulated IGF2BP2 in pancreatic cancer plays a role in cell proliferation [33]. In addition, SNPs of IGF2BP2 and IGF2BP3 has been proved to promote the lymph node metastasis of esophagogastric junction adenocarcinoma [34]. In our GO and KEGG analysis, we noticed that IGF2BP2-related module was correlated with central carbon metabolism, thyroid hormone synthesis and HIF-1signaling pathway, which provided some potential regulatory metabolism of m^6^A RNA modification. In addition to thyroid gland, IGF2BP2 also showed high expression level in lung, small intestine and bone marrow, but the expression of IGF2BP2 did not have great difference between male and female. The next step is to analyze the expression pattern of IGF2BP2 in other organs and explore the potential relationship between IGF2BP2 and regulatory singling pathways, such as HIF-1 signaling pathway.

Furthermore, we explored and validated the prognostic value of m^6^A RNA methylation regulators in PTC. Although FTO, RBM15, YTHDF3, and KIAA1429 were correlated with OS of PTC, we chose IGF2BP2 for deeper analysis as it is the only gene which was correlated with DFS of PTC. We used WGCNA, univariant and multivariant CoxPH to select candidate genes (IGF2BP2, STT3A, MTHFD1 and GSTM4) for construction of a m^6^A-related signature. With the exception of IGF2BP2, other three genes were down-regulated in PTC, and their roles in tumorigenesis have been reported in previous researches. It is well-known that STT3A acts as an enzyme which catalyzes PD-L1 glycosylation and maintain PD-L1 stability, resulting in killing T cells [35]. As a result, low expression level of STT3A can support the immune activity in thyroid cancer tissue, which increases inflammatory mediators, cytokines, chemokines, reactive oxygen species in the tumor immune microenvironment and promotes tumor progression. MTHFD1 and GSTM4 are enzymes of folate metabolism and glutathione metabolism, and both of them have been reported to be related to immunodeficiency and tumor [36, 37]. After K-M plot, ROC curve, univariant and multivariant analyses, this signature showed its great value in predicting DFS of patients with PTC. The result was validated in different cohort (total TCGA cohort, training set and testing set) to ensure the accuracy. We can also notice that risk scores were correlated with T and N classifications, new tumor event and extrathyroidal extension of PTC. These clinical phenotypes were considered to indicators of recurrence and lymph node metastasis, both of which were regarded as determinants of DFS and particularly contribute to the exacerbation of PTC. We also provided a nomogram that reduce the m^6^A-related signature into a single numerical estimate of the probability of an event, such as death, 1-, 3-, 5-year DFS and recurrence, predicting the prognosis of every individual patient. For further study, we prepare to evaluate the clinical prognostic value of this signature by applying to patients who are not limited to internet databases. To deeply explore the mechanism of m^6^A modification, cell and animal experiments are urgently needed to search for downstream target of m^6^A RNA methylation regulators.

## Conclusions

In this study, we performed a comprehensive evaluation of the landscape of m^6^A RNA methylation in PTC by analyzing the RNA expression level, CNVs, SNPs and correlated clinical phenotypes of 19 m^6^A RNA methylation regulators. In NMF clustering analysis, we found that cluster1 and cluster 2 were significantly different in DFS, stage and age, suggesting the important role of m^6^A modification in PTC. After WGCNA, univariant and multivariant CoxPH, IGF2BP2, STT3A, MTHFD1 and GSTM4 were used as candidates for construction of a m^6^A-related signature. This signature was capable to predict the DFS of Patients in different cohort and served as an independent prognostic factor for PTC. It was also correlated with T and N classifications, new tumor event and extrathyroidal extension of PTC. To sum up, IGF2BP2 is a possible biomarker for diagnosis and prognosis of PTC and our m^6^A-related signature is of great significance in predicting DFS of PTC patients.

## Supplementary information

**Additional file 1: Table S1.** The list of antibodies in the Human Protein Atlas.

**Additional file 2: Table S2.** The Mann–Whitney test of differential expressed m^6^A RNA methylation regulators in PTC.

**Additional file 3: Table S3.** Validation of differential expressed m^6^A RNA methylation regulators by GEO database.

**Additional file 4: Figure S1.** Validation of differential expressed m^6^A RNA methylation regulators by Oncomine database.

**Additional file 5: Figure S2.** Validation of differential expressed m^6^A RNA methylation regulators by IHC samples obtained from the Human protein atlas.

**Additional file 6: Table S4.** Using the Chi square test to compare CNVs of m^6^A RNA methylation regulators in normal and tumor tissues.

**Additional file 7: Table S5.** The Mann–Whitney test of differential expressed m^6^A RNA methylation regulators cluster 1 and cluster 2.

**Additional file 8: Figure S3.** The establishment of a gene co-expression network. (A-B) Soft-thresholding power analysis was used to obtain the scale-free fit index of network topology. (C) The cluster was based on the transcriptome data from TCGA. The color intensity represents the clinical phenotypes (fustat, futime, TNM classification, stage, age, gender, new-event, new-event time and extrathyroidal extension and IGF2BP2).

**Additional file 9: Table S6.** Univariant CoxPH analysis of genes in the m^6^A-related module from WGCNA.

**Additional file 10: Table S7.** The multivariate Cox coefficients of MTHFD1, IGF2BP2, STT3A and GSTM4.

## Data Availability

Additional data not presented in the manuscript can be obtained by contacting the authors.

## Abbreviations

PTC: Papillary thyroid cancer
m6A: N^6^-methyladenosine
SNP: Single nucleotide polymorphism
CNV: Copy number variation
OS: Overall survival
DFS: Disease-free survival
WGCNA: Weighted correlation network analysis
CoxPH: Cox proportional hazard model
FTC: Follicular thyroid cancer
MTC: Medullary thyroid cancer
3ʹUTRs: 3ʹ Untranslated regions
TSH: Thyroid stimulating hormone
TCGA: The Cancer Genome Atlas
GEO: Gene expression omnibus
MAF: Mutation annotation format
KEGG: Kyoto Encyclopedia of Genes and Gene Ontology
GO: Gene ontology
K-M: Kaplan–Meier
ROC: Receiver operating characteristic
IGF2BP2: Insulin-like growth factor 2 mRNA binding protein 2

## References

[1] Cabanillas ME, McFadden DG, Durante C (2016). Thyroid cancer. Lancet.

[2] Kitahara CM, Schneider AB, Brenner AV (2017). Thyroid cancer schottenfeld and fraumeni cancer epidemiology and prevention.

[3] Schneider DF, Chen H (2013). New developments in the diagnosis and treatment of thyroid cancer. CA Cancer J Clin.

[4] Elisei R (2018). Thyroid carcinoma. encyclopedia of endocrine diseases.

[5] Wada N, Sugino K, Mimura T, Nagahama M, Kitagawa W, Shibuya H (2009). Pediatric differentiated thyroid carcinoma in stage I: risk factor analysis for disease free survival. BMC Cancer..

[6] Cedar H, Bergman Y (2009). Linking DNA methylation and histone modification: patterns and paradigms. Nat Rev Genet.

[7] Fu Y, Dominissini D, Rechavi G, He C (2014). Gene expression regulation mediated through reversible m6A RNA methylation. Nat Rev Genet.

[8] Bi Z, Liu Y, Zhao Y, Yao Y, Wu R, Liu Q (2019). A dynamic reversible RNA N 6-methyladenosine modification: current status and perspectives. J Cell Physiol.

[9] Meyer KD, Saletore Y, Zumbo P, Elemento O, Mason CE, Jaffrey SR (2012). Comprehensive analysis of mRNA methylation reveals enrichment in 3′ UTRs and near stop codons. Cell.

[10] Meyer KD, Patil DP, Zhou J, Zinoviev A, Skabkin MA, Elemento O (2015). 5′ UTR m6A promotes cap-independent translation. Cell.

[11] Meyer KD, Jaffrey SR (2017). Rethinking m 6 a readers, writers, and erasers. Annu Rev Cell Dev Biol.

[12] Niu Y, Lin Z, Wan A, Chen H, Liang H, Sun L (2019). RNA N6-methyladenosine demethylase FTO promotes breast tumor progression through inhibiting BNIP3. Mol Cancer..

[13] Cui Q, Shi H, Ye P, Li L, Qu Q, Sun G (2017). m 6 A RNA methylation regulates the self-renewal and tumorigenesis of glioblastoma stem cells. Cell Rep..

[14] Zhou J, Wang J, Hong B, Ma K, Xie H, Li L (2019). Gene signatures and prognostic values of m6A regulators in clear cell renal cell carcinoma—a retrospective study using TCGA database. Aging (Albany NY)..

[15] Li Z, Li F, Peng Y, Fang J, Zhou J (2020). Identification of three m6A-related mRNAs signature and risk score for the prognostication of hepatocellular carcinoma. Cancer Med.

[16] Li Y, Xiao J, Bai J, Tian Y, Qu Y, Chen X (2019). Molecular characterization and clinical relevance of m6A regulators across 33 cancer types. Mol Cancer..

[17] Li T, Hu P-S, Zuo Z, Lin J-F, Li X, Wu Q-N (2019). METTL3 facilitates tumor progression via an m6A-IGF2BP2-dependent mechanism in colorectal carcinoma. Mol Cancer..

[18] Chen Y, Peng C, Chen J, Chen D, Yang B, He B (2019). WTAP facilitates progression of hepatocellular carcinoma via m6A-HuR-dependent epigenetic silencing of ETS1. Mol Cancer..

[19] Mayakonda A, Lin D-C, Assenov Y, Plass C, Koeffler HP (2018). Maftools: efficient and comprehensive analysis of somatic variants in cancer. Genome Res.

[20] Asplund A, Edqvist PHD, Schwenk JM, Pontén F (2012). Antibodies for profiling the human proteome-the human protein atlas as a resource for cancer research. Proteomics.

[21] Brunet J-P, Tamayo P, Golub TR, Mesirov JP (2004). Metagenes and molecular pattern discovery using matrix factorization. Proc Natl Acad Sci.

[22] Gaujoux R, Seoighe C (2010). A flexible R package for nonnegative matrix factorization. BMC Bioinf.

[23] Langfelder P, Horvath S (2008). WGCNA: an R package for weighted correlation network analysis. BMC Bioinformatics.

[24] Fiore E, Vitti P (2012). Serum TSH and risk of papillary thyroid cancer in nodular thyroid disease. J Clin Endocrinol Metab.

[25] Zou M, Baitei EY, Alzahrani AS, BinHumaid FS, Alkhafaji D, Al-Rijjal RA (2014). Concomitant RAS, RET/PTC, or BRAF mutations in advanced stage of papillary thyroid carcinoma. Thyroid..

[26] Xing M (2005). BRAF mutation in thyroid cancer. Endocr Relat Cancer.

[27] Barbie DA, Tamayo P, Boehm JS, Kim SY, Moody SE, Dunn IF (2009). Systematic RNA interference reveals that oncogenic KRAS-driven cancers require TBK1. Nature.

[28] Zhao X, Cui L (2019). Development and validation of a m6A RNA methylation regulators-based signature for predicting the prognosis of head and neck squamous cell carcinoma. Am J Cancer Res..

[29] Hong K (2018). Emerging function of N6-methyladenosine in cancer (Review). Oncol Lett..

[30] Liu G, Zhu T, Cui Y, Liu J, Liu J, Zhao Q (2015). Correlation between IGF2BP2 gene polymorphism and the risk of breast cancer in Chinese Han women. Biomed Pharmacother.

[31] He X, Li W, Liang X, Zhu X, Zhang L, Huang Y (2018). IGF2BP2 overexpression indicates poor survival in patients with acute myelocytic leukemia. Cell Physiol Biochem.

[32] Wang K, Jiang L, Zhang Y, Chen C (2020). Progression of thyroid carcinoma is promoted by the m6A methyltransferase METTL3 through regulating m6A methylation on TCF1. Onco Targets Ther..

[33] Xu X, Yu Y, Zong K, Lv P, Gu Y (2019). Up-regulation of IGF2BP2 by multiple mechanisms in pancreatic cancer promotes cancer proliferation by activating the PI3K/Akt signaling pathway. J Exp Clin Cancer Res..

[34] Chen S, Qiu H, Liu C, Wang Y, Tang W, Kang M (2018). Relationship between IGF2BP2 and IGFBP3 polymorphisms and susceptibility to non-small-cell lung cancer: a case–control study in Eastern Chinese Han population. Cancer Manag Res..

[35] Chan L-C, Li C-W, Xia W, Hsu J-M, Lee H-H, Cha J-H (2019). IL-6/JAK1 pathway drives PD-L1 Y112 phosphorylation to promote cancer immune evasion. J Clin Invest..

[36] Sdelci S, Rendeiro AF, Rathert P, You W, Lin JMG, Ringler A (2019). MTHFD1 interaction with BRD4 links folate metabolism to transcriptional regulation. Nat Genet.

[37] Moyer AM, Sun Z, Batzler AJ, Li L, Schaid DJ, Yang P (2010). Glutathione pathway genetic polymorphisms and lung cancer survival after platinum-based chemotherapy. Cancer Epidemiol Biomarkers Prev.

