# Supplementary material for: Identification and validation of m6A RNA methylation regulators with clinical prognostic value in Papillary thyroid cancer
Source: Cancer Cell Int. 2020 May 29;20:203. doi: 10.1186/s12935-020-01283-y (PMC7260751; doi:10.1186/s12935-020-01283-y)
Supplement: Supplementary file 1 — Additional file 1: Table S1. The list of antibodies in the Human Protein Atlas. [file 12935_2020_1283_MOESM1_ESM.docx]

**Table S1 The list of antibodies in the Human Protein Atlas.**

| Antibody ID | Antibody name | Vendor | Cat number |
| --- | --- | --- | --- |
| AB 1850461 | Anti-ALKBH5 antibody produced in rabbit | Sigma-Aldrich | HPA007196 |
| AB 1851480 | Anti-IGF2BP1 antibody produced in rabbit | Sigma-Aldrich | HPA021367 |
| AB 2674491 | Anti-IGF2BP2 antibody produced in rabbit | Sigma-Aldrich | HPA035145 |
| AB 1851483 | Anti-IGF2BP3 antibody produced in rabbit | Sigma-Aldrich | HPA002037 |
| AB 10671965 | Anti-RBM15B antibody produced in rabbit | Sigma-Aldrich | HPA036645 |
| AB 2677293 | Anti-FTO antibody produced in rabbit | Sigma-Aldrich | HPA041086 |
| AB 10600818 | Anti-KIAA1429 antibody produced in rabbit | Sigma-Aldrich | HPA031530 |
| AB 1858866 | Anti-WTAP antibody produced in rabbit | Sigma-Aldrich | HPA010550 |
| AB 2681335 | Anti-HNRNPC antibody produced in rabbit | Sigma-Aldrich | HPA051075 |
| AB 1850836 | Anti-HNRNPA1B1 antibody produced in rabbit | Sigma-Aldrich | HPA001666 |
| AB 10672401 | Anti-METTL14 antibody produced in rabbit | Sigma-Aldrich | HPA038002 |
| AB 1853828 | Anti-METTL16 antibody produced in rabbit | Sigma-Aldrich | HPA020352 |
| AB 10669793 | Anti-YTHDC1 antibody produced in rabbit | Sigma-Aldrich | HPA036462 |
| AB 10672788 | Anti-YTHDC2 antibody produced in rabbit | Sigma-Aldrich | HPA037364 |
| AB 1856113 | Anti-RBM15 antibody produced in rabbit | Sigma-Aldrich | HPA019824 |
