# Supplementary material for: Identification and validation of m6A RNA methylation regulators with clinical prognostic value in Papillary thyroid cancer
Source: Cancer Cell Int. 2020 May 29;20:203. doi: 10.1186/s12935-020-01283-y (PMC7260751; doi:10.1186/s12935-020-01283-y)
Supplement: Supplementary file 2 — Additional file 2: Table S2. The Mann–Whitney test of differential expressed m6A RNA methylation regulators in PTC. [file 12935_2020_1283_MOESM2_ESM.docx]

**Table S2 The Mann-Whitney test of differential expressed m6A RNA methylation regulators in PTC.**

| Gene | conMean | treatMean | logFC | P Value |
| --- | --- | --- | --- | --- |
| METTL3 | 7.094791 | 6.126893 | -0.2116 | 7.20E-05 |
| YTHDC1 | 16.47524 | 12.34067 | -0.41688 | 3.72E-19 |
| FTO | 5.22394 | 3.931592 | -0.41002 | 7.38E-16 |
| METTL14 | 5.2414 | 3.955608 | -0.40605 | 2.07E-19 |
| RBM15 | 2.681662 | 1.84282 | -0.54121 | 9.48E-24 |
| IGF2BP2 | 3.425883 | 12.2507 | 1.838316 | 1.79E-24 |
| IGF2BP1 | 0.014391 | 0.014169 | -0.02245 | 1.93E-07 |
| YTHDF3 | 12.07129 | 10.1966 | -0.24349 | 4.01E-08 |
| WTAP | 17.36785 | 14.4864 | -0.26172 | 1.42E-09 |
| HNRNPA2B1 | 93.17717 | 79.45649 | -0.22981 | 1.04E-09 |
| HNRNPC | 44.56087 | 48.67938 | 0.127533 | 6.82E-05 |
| ALKBH5 | 38.92789 | 34.66377 | -0.16738 | 5.13E-08 |
| YTHDF2 | 16.99332 | 16.94579 | -0.00404 | 0.87885 |
| METTL16 | 5.938268 | 5.639443 | -0.07449 | 0.021783 |
| YTHDC2 | 3.200695 | 2.698562 | -0.24619 | 1.87E-06 |
| KIAA1429 | 5.489613 | 4.713165 | -0.22001 | 4.31E-08 |
| IGF2BP3 | 0.139348 | 0.255979 | 0.877334 | 2.29E-10 |
| RBM15B | 11.01082 | 11.69087 | 0.08646 | 0.003616 |
| YTHDF1 | 22.62329 | 20.70566 | -0.12778 | 2.53E-06 |
