# Supplementary material for: Identification and validation of m6A RNA methylation regulators with clinical prognostic value in Papillary thyroid cancer
Source: Cancer Cell Int. 2020 May 29;20:203. doi: 10.1186/s12935-020-01283-y (PMC7260751; doi:10.1186/s12935-020-01283-y)
Supplement: Supplementary file 3 — Additional file 3: Table S3. Validation of differential expressed m6A RNA methylation regulators by GEO database. [file 12935_2020_1283_MOESM3_ESM.docx]

**Table S3 Validation of differential expressed m6A RNA methylation regulators by GEO database.**

| Gene | logFC | AveExpr | P Value |
| --- | --- | --- | --- |
| IGF2BP2 | 2.31287 | 5.166205 | **1.50E-10** |
| RBM15 | -0.58603 | 6.785079 | **8.76E-05** |
| FTO | -0.42057 | 8.238309 | **0.000581** |
| YTHDC1 | -0.31548 | 6.044922 | **0.000704** |
| RBM15B | 0.176994 | 7.485538 | **0.00382** |
| YTHDF3 | -0.36733 | 7.851216 | **0.005467** |
| HNRNPC | -0.23023 | 9.274222 | **0.008578** |
| HNRNPA2B1 | -0.16248 | 11.24731 | **0.035587** |
| YTHDF1 | -0.20762 | 8.472431 | **0.04697** |
| METTL3 | -0.27495 | 6.433121 | 0.130517 |
| WTAP | -0.11801 | 6.231295 | 0.156297 |
| YTHDC2 | -0.13614 | 4.006152 | 0.214492 |
| IGF2BP3 | -0.07204 | 2.305732 | 0.232607 |
| YTHDF2 | -0.03096 | 8.450459 | 0.80763 |
