# Supplementary figures and images for: Identification and validation of m6A RNA methylation regulators with clinical prognostic value in Papillary thyroid cancer
Source: Cancer Cell Int. 2020 May 29;20:203. doi: 10.1186/s12935-020-01283-y (PMC7260751; doi:10.1186/s12935-020-01283-y)

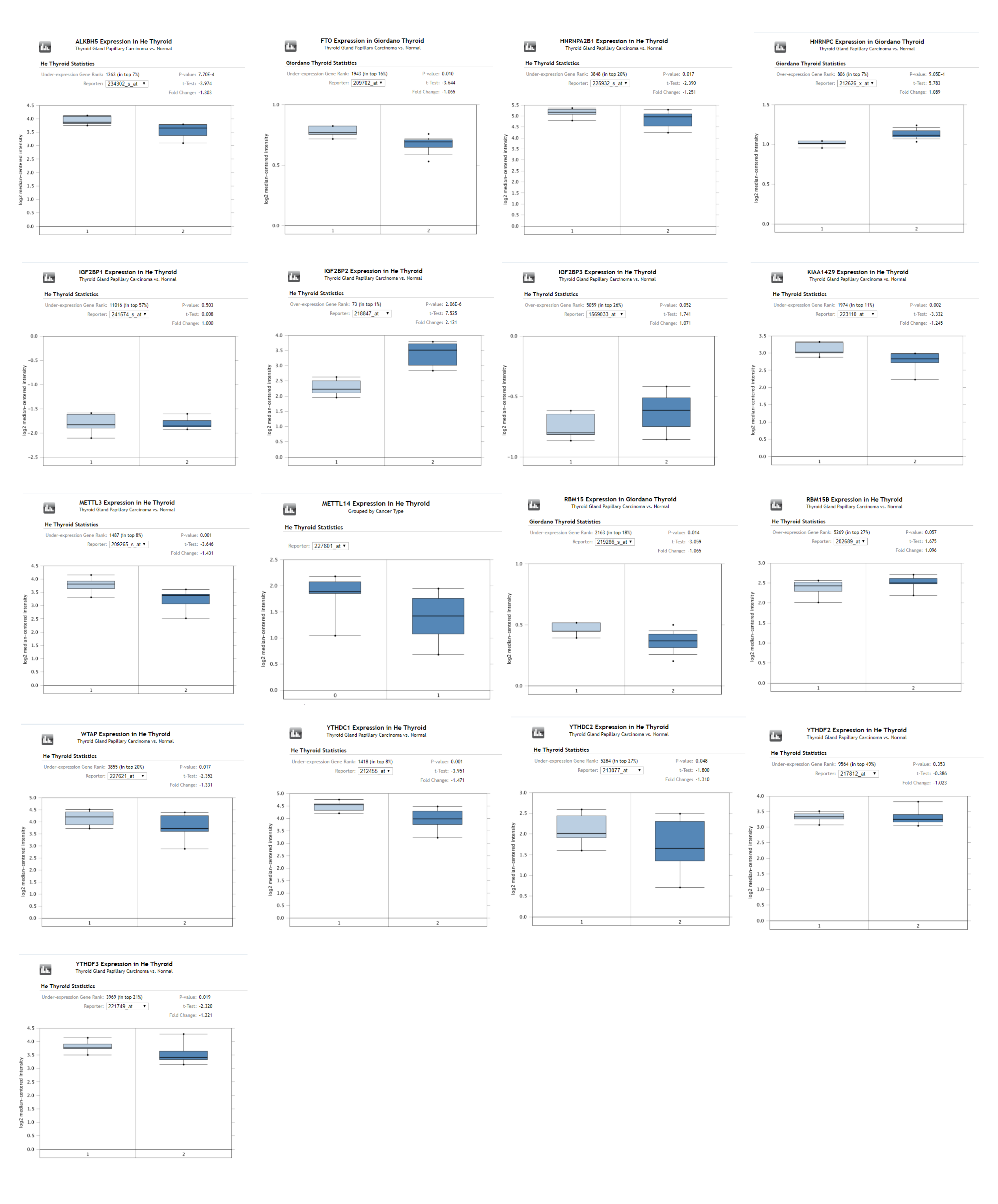

Supplement: Supplementary file 4 — Additional file 4: Figure S1. Validation of differential expressed m6A RNA methylation regulators by Oncomine database. [file 12935_2020_1283_MOESM4_ESM.tif]

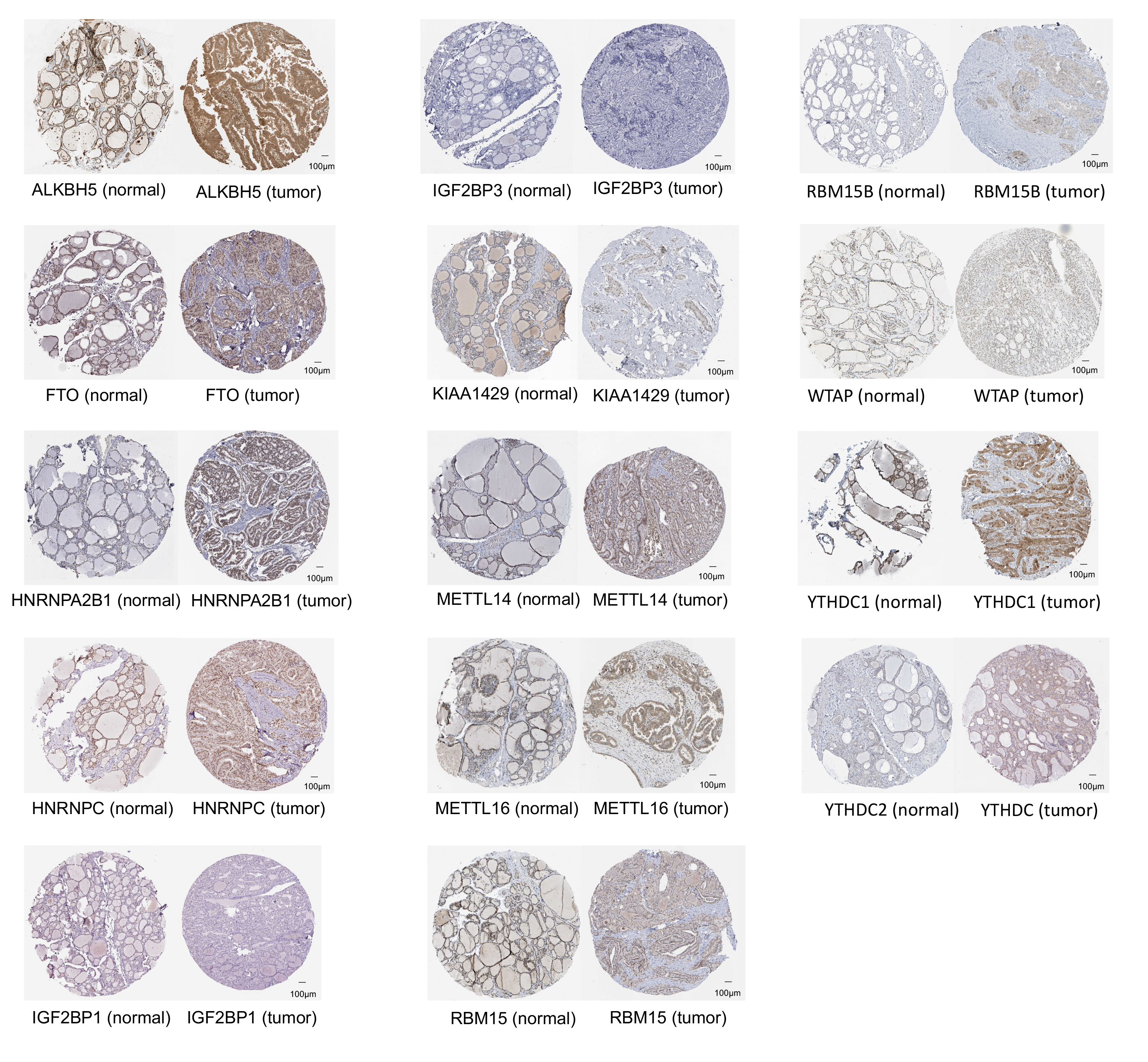

Supplement: Supplementary file 5 — Additional file 5: Figure S2. Validation of differential expressed m6A RNA methylation regulators by IHC samples obtained from the Human protein atlas. [file 12935_2020_1283_MOESM5_ESM.tif]

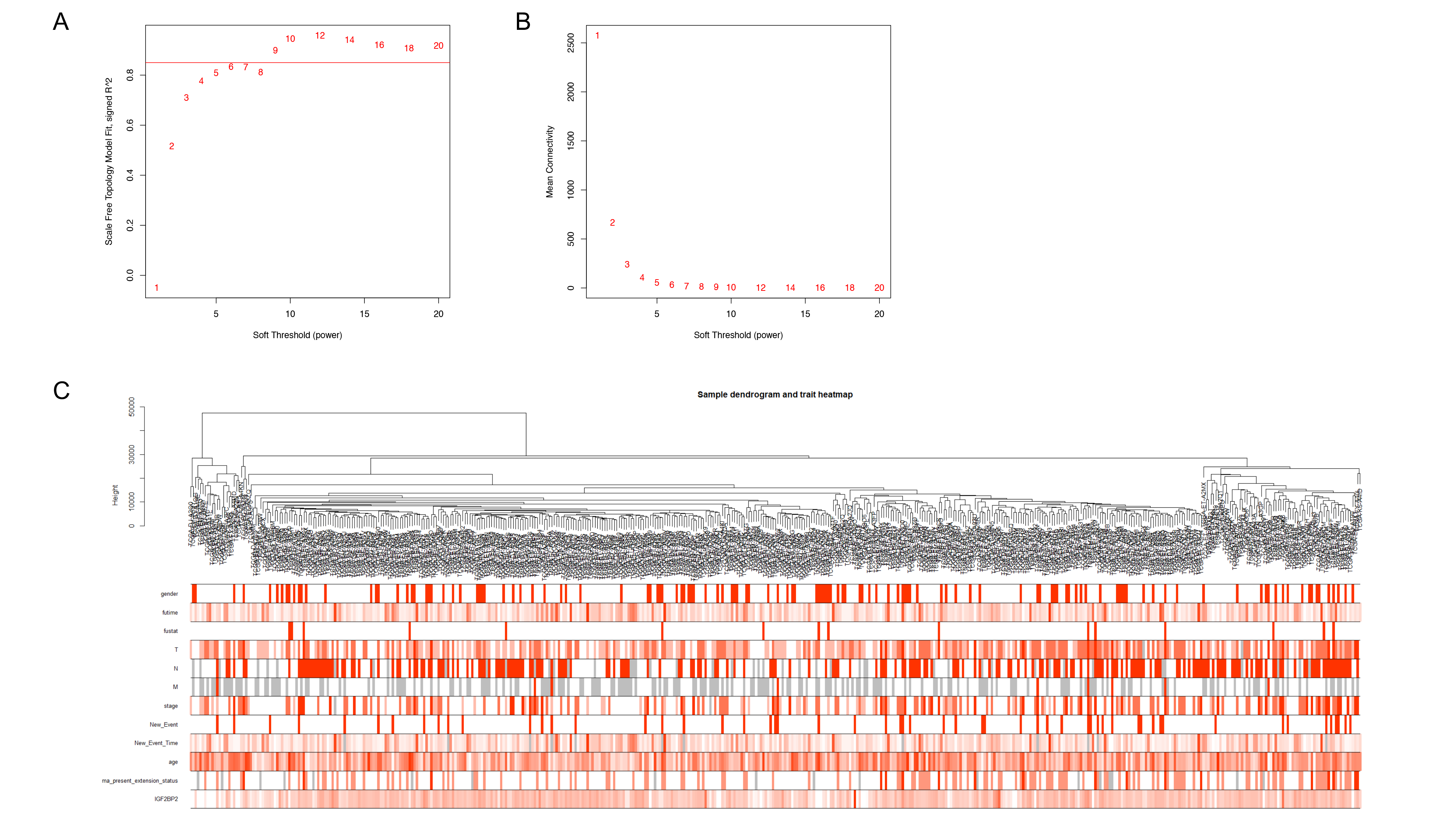

Supplement: Supplementary file 8 — Additional file 8: Figure S3. The establishment of a gene co-expression network. (A-B) Soft-thresholding power analysis was used to obtain the scale-free fit index of network topology. (C) The cluster was based on the transcriptome data from TCGA. The color intensity represents the clinical phenotypes (fustat, futime, TNM classification, stage, age, gender, new-event, new-event time and extrathyroidal extension and IGF2BP2). [file 12935_2020_1283_MOESM8_ESM.tif]
