# Supplementary material for: Identification and validation of m6A RNA methylation regulators with clinical prognostic value in Papillary thyroid cancer
Source: Cancer Cell Int. 2020 May 29;20:203. doi: 10.1186/s12935-020-01283-y (PMC7260751; doi:10.1186/s12935-020-01283-y)
Supplement: Supplementary file 6 — Additional file 6: Table S4. Using the Chi square test to compare CNVs of m6A RNA methylation regulators in normal and tumor tissues. [file 12935_2020_1283_MOESM6_ESM.docx]

**Table S4** **Using the chi-square test to compare CNVs of m6A RNA methylation regulators in normal and tumor tissues.**

| HNRNPA2B1 | Normal | Tumor | P value |
| --- | --- | --- | --- |
| Without CNV | 472 | 480 | **0.005079** |
| With CNV | 0 | 10 |  |
| IGF2BP3 | Normal | Tumor | P value |
| Without CNV | 472 | 480 | **0.005079** |
| With CNV | 0 | 10 |  |
| YTHDC2 | Normal | Tumor | P value |
| Without CNV | 470 | 482 | 0.125965 |
| With CNV | 2 | 8 |  |
| METTL16 | Normal | Tumor | P value |
| Without CNV | 472 | 484 | **0.045284** |
| With CNV | 0 | 6 |  |
| IGF2BP1 | Normal | Tumor | P value |
| Without CNV | 470 | 485 | 0.478242 |
| With CNV | 2 | 5 |  |
| ALKBH5 | Normal | Tumor | P value |
| Without CNV | 472 | 485 | 0.079794 |
| With CNV | 0 | 5 |  |
| WTAP | Normal | Tumor | P value |
| Without CNV | 472 | 487 | 0.26091 |
| With CNV | 0 | 3 |  |
| FTO | Normal | Tumor | P value |
| Without CNV | 471 | 487 | 0.642917 |
| With CNV | 1 | 3 |  |
| YTHDF1 | Normal | Tumor | P value |
| Without CNV | 472 | 487 | 0.26091 |
| With CNV | 0 | 3 |  |
| METTL14 | Normal | Tumor | P value |
| Without CNV | 471 | 488 | 1 |
| With CNV | 1 | 2 |  |
| YTHDF2 | Normal | Tumor | P value |
| Without CNV | 472 | 489 | 1 |
| With CNV | 0 | 1 |  |
| RBM15 | Normal | Tumor | P value |
| Without CNV | 472 | 489 | 1 |
| With CNV | 0 | 1 |  |
| YTHDC1 | Normal | Tumor | P value |
| Without CNV | 472 | 489 | 1 |
| With CNV | 0 | 1 |  |
