# Supplementary material for: Identification and validation of m6A RNA methylation regulators with clinical prognostic value in Papillary thyroid cancer
Source: Cancer Cell Int. 2020 May 29;20:203. doi: 10.1186/s12935-020-01283-y (PMC7260751; doi:10.1186/s12935-020-01283-y)
Supplement: Supplementary file 7 — Additional file 7: Table S5. The Mann–Whitney test of differential expressed m6A RNA methylation regulators cluster 1 and cluster 2. [file 12935_2020_1283_MOESM7_ESM.docx]

**Table S5 The Mann-Whitney test of differential expressed m6A RNA methylation regulators cluster 1 and cluster 2.**

| Gene | Cluster 1 | Cluster 2 | logFC(cluster2/cluster1) | P Value |
| --- | --- | --- | --- | --- |
| IGF2BP2 | 7.797334 | 4.688511 | -0.73385 | 1.25E-25 |
| HNRNPC | 26.29322 | 21.85248 | -0.26689 | 9.02E-15 |
| ALKBH5 | 16.552 | 20.41776 | 0.302819 | 2.93E-14 |
| IGF2BP3 | 0.52569 | 1.391907 | 1.404778 | 6.22E-09 |
| FTO | 2.347093 | 3.180063 | 0.43818 | 2.14E-06 |
| RBM15B | 6.517989 | 6.393818 | -0.02775 | 1.47E-05 |
| WTAP | 7.924204 | 7.963015 | 0.007049 | 0.019528 |
| IGF2BP1 | 0.508362 | 1.01029 | 0.990843 | 0.038684 |
| METTL3 | 3.642563 | 3.852128 | 0.080702 | 0.062373 |
| YTHDC1 | 6.796484 | 6.942703 | 0.030709 | 0.094964 |
| RBM15 | 1.402981 | 1.94981 | 0.474838 | 0.11645 |
| HNRNPA2B1 | 41.0085 | 39.50223 | -0.05399 | 0.156202 |
| YTHDF1 | 10.64493 | 11.60562 | 0.124657 | 0.166976 |
| METTL14 | 2.493693 | 2.922292 | 0.228816 | 0.332899 |
| KIAA1429 | 2.83729 | 3.264154 | 0.202196 | 0.552135 |
| YTHDF3 | 5.515333 | 5.865718 | 0.08886 | 0.663794 |
| YTHDC2 | 1.864644 | 2.297317 | 0.30105 | 0.701928 |
| METTL16 | 3.290639 | 3.769494 | 0.196003 | 0.783471 |
| YTHDF2 | 8.957589 | 9.403601 | 0.070103 | 0.804275 |
