# Supplementary material for: Identification and validation of m6A RNA methylation regulators with clinical prognostic value in Papillary thyroid cancer
Source: Cancer Cell Int. 2020 May 29;20:203. doi: 10.1186/s12935-020-01283-y (PMC7260751; doi:10.1186/s12935-020-01283-y)
Supplement: Supplementary file 9 — Additional file 9: Table S6. Univariant CoxPH analysis of genes in the m6A-related module from WGCNA. [file 12935_2020_1283_MOESM9_ESM.docx]

**Table S6 Univariant CoxPH analysis of genes in the m6A-related module from WGCNA.**

| Gene | HR | HR.95L | HR.95H | P-value |
| --- | --- | --- | --- | --- |
| IGF2BP2 | 1.185141 | 1.08418 | 1.295503 | **0.000185** |
| STT3A | 0.890513 | 0.80036 | 0.990822 | **0.033232** |
| MTHFD1 | 1.317577 | 1.00767 | 1.722795 | **0.04382** |
| GSTM4 | 1.12326 | 1.00073 | 1.260793 | **0.048569** |
| ETFA | 0.796982 | 0.628494 | 1.010639 | 0.061119 |
| TCEANC | 0.098472 | 0.007541 | 1.285784 | 0.077025 |
| PGAM5 | 1.19689 | 0.980526 | 1.460997 | 0.077286 |
| TMX2 | 0.938255 | 0.874175 | 1.007032 | 0.077428 |
| LRRIQ3 | 0.000431 | 5.12E-08 | 3.631419 | 0.092894 |
| BSPRY | 1.032224 | 0.994114 | 1.071795 | 0.098454 |
| SRPRB | 0.877579 | 0.751402 | 1.024943 | 0.099172 |
| CTIF | 0.666796 | 0.411633 | 1.080129 | 0.099608 |
| TRIM50 | 1.588494 | 0.909498 | 2.774403 | 0.103832 |
| SNRK | 1.112159 | 0.977314 | 1.265609 | 0.106963 |
| NUPR1 | 1.006261 | 0.998637 | 1.013944 | 0.107732 |
| RCBTB2 | 0.868695 | 0.730062 | 1.033653 | 0.112551 |
| TMEM233 | 1.021578 | 0.994892 | 1.04898 | 0.11393 |
| SFXN4 | 0.887492 | 0.765075 | 1.029496 | 0.115003 |
| CUX2 | 1.105642 | 0.975461 | 1.253196 | 0.116126 |
| PTPRE | 1.084602 | 0.975259 | 1.206203 | 0.13416 |
| COPZ2 | 0.899222 | 0.782401 | 1.033486 | 0.134635 |
| ADAM22 | 0.217695 | 0.029173 | 1.624465 | 0.137061 |
| HYOU1 | 0.968647 | 0.927794 | 1.011298 | 0.147348 |
| MCCC1 | 1.204931 | 0.934065 | 1.554344 | 0.151303 |
| TMCO1 | 0.902879 | 0.78483 | 1.038685 | 0.152988 |
| SYBU | 0.722726 | 0.46185 | 1.13096 | 0.155228 |
| TIMM21 | 0.594845 | 0.290298 | 1.218888 | 0.155846 |
| ABHD6 | 0.704727 | 0.43261 | 1.148008 | 0.159853 |
| DCLRE1A | 1.479113 | 0.852227 | 2.567129 | 0.164065 |
| COQ7 | 1.252131 | 0.911652 | 1.71977 | 0.164928 |
| IQCH | 0.224971 | 0.026705 | 1.895229 | 0.170072 |
| MAPK9 | 1.223408 | 0.914309 | 1.637003 | 0.174767 |
| FDX1 | 0.878138 | 0.727415 | 1.06009 | 0.176186 |
| DLST | 1.052325 | 0.977257 | 1.13316 | 0.176794 |
| COX5A | 0.973388 | 0.935986 | 1.012284 | 0.177257 |
| CLCNKB | 0.77718 | 0.5379 | 1.122903 | 0.179404 |
| LDHB | 0.98935 | 0.974002 | 1.004941 | 0.17955 |
| VDAC1 | 1.046114 | 0.978718 | 1.118151 | 0.184558 |
| PDHX | 1.334112 | 0.870122 | 2.045524 | 0.186181 |
| MMACHC | 0.408755 | 0.108216 | 1.543956 | 0.187037 |
| ECE1 | 1.009404 | 0.995423 | 1.02358 | 0.188402 |
| SIRT5 | 0.641521 | 0.330595 | 1.244874 | 0.189384 |
| LIN7A | 0.449534 | 0.135995 | 1.485948 | 0.189957 |
| SFXN3 | 1.113151 | 0.947333 | 1.307994 | 0.192733 |
| COX7B | 0.951899 | 0.883723 | 1.025335 | 0.193564 |
| HS6ST3 | 0.323494 | 0.058742 | 1.781497 | 0.194783 |
| AC245041.1 | 4.39E-31 | 5.28E-77 | 3.65E+15 | 0.195074 |
| CHCHD3 | 1.061714 | 0.969741 | 1.162411 | 0.195205 |
| OR2W3 | 0.802308 | 0.574884 | 1.1197 | 0.195265 |
| PDLIM7 | 0.928872 | 0.830124 | 1.039367 | 0.198218 |
| ALG12 | 0.744054 | 0.473191 | 1.169965 | 0.200468 |
| MLNR | 0.001949 | 1.38E-07 | 27.52162 | 0.200544 |
| SERTM1 | 0.632791 | 0.313755 | 1.27623 | 0.201069 |
| ZNF684 | 0.328122 | 0.058001 | 1.856255 | 0.207538 |
| LASP1 | 1.037198 | 0.979877 | 1.097872 | 0.207982 |
| RBPMS2 | 0.883699 | 0.728334 | 1.072206 | 0.21011 |
| GRPEL1 | 0.719089 | 0.426358 | 1.212805 | 0.216265 |
| WDFY2 | 0.473941 | 0.144699 | 1.552329 | 0.217392 |
| ATP5G3 | 0.934546 | 0.838808 | 1.041211 | 0.219596 |
| OXSM | 0.719452 | 0.425085 | 1.217667 | 0.220038 |
| TMEM99 | 0.842304 | 0.639403 | 1.109591 | 0.222302 |
| ACAD8 | 1.06087 | 0.964392 | 1.167 | 0.224496 |
| SNX11 | 0.866956 | 0.687991 | 1.092475 | 0.226194 |
| CRYAB | 0.988881 | 0.971049 | 1.007041 | 0.22849 |
| CRADD | 0.597484 | 0.258226 | 1.38246 | 0.228862 |
| RIN1 | 0.714913 | 0.412104 | 1.240222 | 0.23248 |
| SLC43A1 | 0.790727 | 0.537104 | 1.164111 | 0.234088 |
| RBPMS | 0.9344 | 0.83496 | 1.045684 | 0.237262 |
| CIPC | 1.102073 | 0.937858 | 1.295043 | 0.23775 |
| APOO | 0.894869 | 0.74402 | 1.076302 | 0.238279 |
| TEX2 | 1.035878 | 0.976719 | 1.09862 | 0.240062 |
| FAM124A | 0.334392 | 0.053471 | 2.091174 | 0.241514 |
| EPB41L4B | 1.061988 | 0.960242 | 1.174516 | 0.241827 |
| CANX | 0.993755 | 0.983368 | 1.004252 | 0.242613 |
| ACAD10 | 0.770543 | 0.497482 | 1.193482 | 0.242951 |
| ANKRD9 | 0.684847 | 0.362644 | 1.293322 | 0.2432 |
| RCAN2 | 0.922936 | 0.805576 | 1.057395 | 0.247802 |
| TRAP1 | 1.130821 | 0.918 | 1.39298 | 0.247802 |
| HK1 | 0.960917 | 0.898086 | 1.028144 | 0.247881 |
| ESRRA | 0.921768 | 0.802507 | 1.058754 | 0.249182 |
| SLC25A13 | 1.094817 | 0.938404 | 1.277302 | 0.249446 |
| PDIA6 | 0.987519 | 0.96663 | 1.00886 | 0.249604 |
| UQCC1 | 0.779555 | 0.509411 | 1.192959 | 0.251303 |
| MKKS | 0.780486 | 0.51098 | 1.19214 | 0.251481 |
| TIMM17A | 0.895134 | 0.740236 | 1.082444 | 0.253136 |
| TSPAN33 | 1.01349 | 0.990454 | 1.037062 | 0.253325 |
| MCUR1 | 1.108161 | 0.928763 | 1.322211 | 0.254373 |
| LAMTOR3 | 1.119664 | 0.921594 | 1.360303 | 0.255147 |
| SDHA | 1.06828 | 0.952958 | 1.197557 | 0.257112 |
| NUCB2 | 0.915897 | 0.785913 | 1.067378 | 0.260598 |
| MRPS35 | 1.042811 | 0.968985 | 1.122263 | 0.263154 |
| AMIGO1 | 0.823583 | 0.585796 | 1.157892 | 0.264172 |
| SUCLG2 | 1.036958 | 0.972762 | 1.10539 | 0.2657 |
| GZF1 | 0.708022 | 0.38422 | 1.304711 | 0.268244 |
| SLC5A8 | 0.947351 | 0.860725 | 1.042695 | 0.268965 |
| MIPEP | 1.172195 | 0.884131 | 1.554116 | 0.269538 |
| LIPH | 1.024114 | 0.981388 | 1.068701 | 0.273129 |
| ATG4A | 0.836887 | 0.608457 | 1.151075 | 0.273574 |
| CDKL1 | 2.051532 | 0.565537 | 7.442098 | 0.274393 |
| GBA2 | 0.91928 | 0.790199 | 1.069446 | 0.275605 |
| LRP2 | 0.955071 | 0.87913 | 1.037573 | 0.276843 |
| MTFR1L | 0.904514 | 0.754786 | 1.083944 | 0.277064 |
| DCAF4 | 1.225651 | 0.848415 | 1.770619 | 0.278316 |
| ZNF664 | 1.015287 | 0.987678 | 1.043668 | 0.280779 |
| GALE | 1.02924 | 0.976536 | 1.084789 | 0.282532 |
| GRB7 | 1.036657 | 0.970479 | 1.107347 | 0.284779 |
| KIF21A | 1.321848 | 0.791857 | 2.206562 | 0.285837 |
| KLLN | 0.173604 | 0.006942 | 4.341659 | 0.286403 |
| RAB3IP | 1.496875 | 0.712912 | 3.142932 | 0.286499 |
| FRK | 0.105831 | 0.001657 | 6.759246 | 0.289618 |
| COA7 | 1.199563 | 0.85583 | 1.681352 | 0.290859 |
| DUSP19 | 0.501988 | 0.139559 | 1.805627 | 0.291327 |
| RNF186 | 78.32405 | 0.023767 | 258117.1 | 0.291353 |
| CKMT2 | 0.841489 | 0.609446 | 1.161881 | 0.294428 |
| CDC14B | 1.214174 | 0.843061 | 1.748649 | 0.297085 |
| APOOL | 1.160852 | 0.873389 | 1.54293 | 0.30421 |
| NDUFA10 | 0.852209 | 0.628096 | 1.156288 | 0.304316 |
| TIMMDC1 | 0.927042 | 0.802259 | 1.071235 | 0.304395 |
| CDADC1 | 0.775206 | 0.476352 | 1.261557 | 0.30545 |
| CCSER1 | 6.421795 | 0.181368 | 227.3805 | 0.306842 |
| PQLC2L | 0.639825 | 0.271572 | 1.507431 | 0.3071 |
| ALDH5A1 | 1.085784 | 0.927125 | 1.271595 | 0.307186 |
| MTFR1 | 0.705568 | 0.361246 | 1.378083 | 0.307228 |
| HLF | 0.909912 | 0.758326 | 1.091799 | 0.309929 |
| PCCA | 0.80598 | 0.531277 | 1.222724 | 0.310417 |
| PARM1 | 1.006887 | 0.993599 | 1.020353 | 0.311252 |
| CDC25A | 1.547802 | 0.662994 | 3.613442 | 0.312564 |
| FHL1 | 0.990505 | 0.97232 | 1.00903 | 0.312904 |
| FASTKD1 | 0.737247 | 0.406923 | 1.335714 | 0.314743 |
| TRIM47 | 1.047679 | 0.956586 | 1.147447 | 0.315567 |
| AGBL5 | 1.128957 | 0.890267 | 1.431641 | 0.316896 |
| 2-Mar | 0.945452 | 0.847092 | 1.055232 | 0.316933 |
| SPAG9 | 1.064214 | 0.941728 | 1.202632 | 0.318474 |
| KCTD17 | 1.083943 | 0.924823 | 1.27044 | 0.319678 |
| H2AFV | 1.033333 | 0.968645 | 1.102342 | 0.320166 |
| SH3BGR | 0.905989 | 0.745207 | 1.10146 | 0.321944 |
| C5orf47 | 48.7609 | 0.021557 | 110296.3 | 0.323982 |
| TMEM171 | 0.925034 | 0.792073 | 1.080315 | 0.325004 |
| SMAD9 | 0.943335 | 0.839868 | 1.059548 | 0.325052 |
| SYPL2 | 0.862473 | 0.640972 | 1.160519 | 0.328591 |
| DTD2 | 0.778929 | 0.471003 | 1.288166 | 0.33036 |
| RAB29 | 1.039284 | 0.961458 | 1.123409 | 0.331923 |
| RWDD4 | 0.78158 | 0.473971 | 1.28883 | 0.334202 |
| SLC25A15 | 0.936076 | 0.818491 | 1.070553 | 0.334779 |
| IYD | 1.001874 | 0.998066 | 1.005697 | 0.335212 |
| TSFM | 1.108761 | 0.898203 | 1.368678 | 0.33664 |
| ALKBH5 | 0.962262 | 0.889616 | 1.040841 | 0.336802 |
| CAVIN1 | 0.99398 | 0.981691 | 1.006422 | 0.341435 |
| TGFB1I1 | 0.904256 | 0.734274 | 1.113589 | 0.343489 |
| MPC1 | 0.964731 | 0.895476 | 1.039342 | 0.344806 |
| IPCEF1 | 0.891768 | 0.702995 | 1.131232 | 0.345221 |
| PMPCB | 1.066514 | 0.932751 | 1.219459 | 0.346296 |
| GPI | 0.958794 | 0.878248 | 1.046727 | 0.347271 |
| MRPS30 | 1.18951 | 0.828202 | 1.708441 | 0.347475 |
| NT5C3B | 0.959651 | 0.880257 | 1.046207 | 0.349917 |
| TMEM267 | 0.906314 | 0.736898 | 1.114681 | 0.351501 |
| COX18 | 1.527206 | 0.624442 | 3.735111 | 0.353418 |
| GPR157 | 1.176492 | 0.833517 | 1.660596 | 0.355304 |
| PHYH | 1.016152 | 0.982195 | 1.051284 | 0.355495 |
| KCNQ1 | 0.983891 | 0.950501 | 1.018455 | 0.356593 |
| SHISA5 | 0.981144 | 0.942094 | 1.021813 | 0.35829 |
| ERO1B | 0.918223 | 0.765385 | 1.10158 | 0.358382 |
| EPN3 | 1.136273 | 0.864681 | 1.493171 | 0.359305 |
| MUT | 1.0906 | 0.905992 | 1.312824 | 0.359358 |
| CCDC28B | 0.868502 | 0.642105 | 1.174724 | 0.360227 |
| SLC33A1 | 0.858588 | 0.619346 | 1.190246 | 0.360248 |
| CYB561 | 0.967102 | 0.900161 | 1.039022 | 0.360708 |
| MED20 | 0.84773 | 0.594684 | 1.208452 | 0.361119 |
| RAVER2 | 1.261426 | 0.763078 | 2.085234 | 0.365148 |
| MRPS14 | 0.900504 | 0.717275 | 1.130541 | 0.366582 |
| CPT2 | 1.082668 | 0.910986 | 1.286705 | 0.367237 |
| QDPR | 0.929776 | 0.793493 | 1.089466 | 0.367923 |
| TMEM254 | 1.060769 | 0.93235 | 1.206876 | 0.370229 |
| QRSL1 | 1.223845 | 0.786497 | 1.904388 | 0.37058 |
| BCL2 | 0.959949 | 0.877766 | 1.049826 | 0.370717 |
| MRO | 0.768552 | 0.430395 | 1.372396 | 0.373532 |
| NDUFS1 | 1.089964 | 0.900446 | 1.319372 | 0.376731 |
| PPARGC1A | 0.897271 | 0.704459 | 1.142856 | 0.379848 |
| RRAGD | 1.019939 | 0.975973 | 1.065886 | 0.379853 |
| SLC18B1 | 0.905454 | 0.725152 | 1.130585 | 0.380681 |
| NATD1 | 1.065533 | 0.924516 | 1.22806 | 0.38083 |
| NDUFB5 | 0.913631 | 0.74593 | 1.119036 | 0.382666 |
| CNNM2 | 0.547073 | 0.140908 | 2.124006 | 0.38347 |
| SDHD | 0.94326 | 0.826244 | 1.076848 | 0.387381 |
| KLK15 | 1.151741 | 0.835843 | 1.587028 | 0.387752 |
| FAHD2A | 1.086694 | 0.899825 | 1.312371 | 0.387824 |
| CTF1 | 0.95899 | 0.871892 | 1.054789 | 0.388708 |
| PIAS2 | 0.622896 | 0.211995 | 1.830227 | 0.38934 |
| RNF157 | 1.119508 | 0.864224 | 1.4502 | 0.392606 |
| DNAJA3 | 1.105671 | 0.878028 | 1.392336 | 0.393079 |
| TRIM58 | 0.913547 | 0.741971 | 1.124798 | 0.394256 |
| OGDHL | 0.958709 | 0.870002 | 1.056461 | 0.394649 |
| CCDC110 | 0.606255 | 0.190186 | 1.932561 | 0.397503 |
| MRPL15 | 0.958163 | 0.867828 | 1.0579 | 0.397604 |
| NLK | 1.089556 | 0.891505 | 1.331604 | 0.402053 |
| SDHB | 1.044939 | 0.942802 | 1.158141 | 0.402239 |
| NDUFS2 | 0.951446 | 0.846438 | 1.069481 | 0.404187 |
| GFM2 | 1.135306 | 0.842157 | 1.530497 | 0.405008 |
| NDUFB3 | 0.969285 | 0.90044 | 1.043393 | 0.406579 |
| SLC25A20 | 0.926583 | 0.773053 | 1.110605 | 0.409386 |
| CLYBL | 0.638739 | 0.216646 | 1.883199 | 0.416467 |
| LARP1 | 1.02241 | 0.969043 | 1.078716 | 0.41778 |
| STRAP | 1.0149 | 0.97915 | 1.051956 | 0.418882 |
| KATNAL2 | 0.84608 | 0.563965 | 1.269318 | 0.419304 |
| GHR | 0.560464 | 0.13625 | 2.305478 | 0.422329 |
| FXYD5 | 0.990981 | 0.969262 | 1.013187 | 0.422967 |
| ALDH9A1 | 0.99091 | 0.968881 | 1.013439 | 0.425963 |
| MYO19 | 1.372997 | 0.62654 | 3.008782 | 0.428401 |
| GNA14 | 0.952883 | 0.845557 | 1.073832 | 0.428593 |
| EBAG9 | 0.932564 | 0.783752 | 1.109631 | 0.431201 |
| ITPR1 | 0.954326 | 0.849362 | 1.072261 | 0.431646 |
| CPB2 | 0.05614 | 4.25E-05 | 74.22154 | 0.43223 |
| MDH2 | 1.011606 | 0.982881 | 1.041171 | 0.432398 |
| SDHAF3 | 0.875291 | 0.626922 | 1.222057 | 0.434066 |
| PPM1L | 0.895023 | 0.677864 | 1.181752 | 0.434107 |
| HCCS | 1.086607 | 0.881755 | 1.339051 | 0.435811 |
| MLEC | 0.989513 | 0.963612 | 1.016111 | 0.435982 |
| STARD13 | 0.909261 | 0.715546 | 1.15542 | 0.436473 |
| RAB19 | 1.221114 | 0.737615 | 2.021541 | 0.437338 |
| NFS1 | 0.75779 | 0.375948 | 1.527459 | 0.438041 |
| AC092718.3 | 1.176315 | 0.780034 | 1.773918 | 0.438485 |
| KIAA1456 | 0.901316 | 0.692961 | 1.172318 | 0.438553 |
| LENG9 | 1.195166 | 0.758958 | 1.882082 | 0.441587 |
| TCTA | 0.975701 | 0.916442 | 1.038792 | 0.441611 |
| SETD3 | 1.035287 | 0.947445 | 1.131274 | 0.44333 |
| GPR61 | 1.252285 | 0.703597 | 2.228858 | 0.44438 |
| FBXO25 | 0.772281 | 0.397452 | 1.500603 | 0.445798 |
| C11orf74 | 0.968409 | 0.891673 | 1.051749 | 0.445995 |
| BCO2 | 0.55168 | 0.119436 | 2.548239 | 0.446155 |
| DIS3L | 0.893596 | 0.668689 | 1.194149 | 0.446949 |
| SIKE1 | 0.8498 | 0.558475 | 1.293093 | 0.447325 |
| HIBADH | 1.008665 | 0.986462 | 1.031367 | 0.447419 |
| ZSCAN5A | 0.285084 | 0.01103 | 7.368246 | 0.449452 |
| RTN3 | 0.985266 | 0.94803 | 1.023963 | 0.450134 |
| ZMAT4 | 0.927366 | 0.762062 | 1.128527 | 0.451557 |
| THNSL1 | 0.858028 | 0.575918 | 1.278328 | 0.45159 |
| MDS2 | 1.325284 | 0.636423 | 2.759767 | 0.451746 |
| KCNIP3 | 0.937161 | 0.791168 | 1.110094 | 0.452569 |
| RAB3A | 0.798048 | 0.44298 | 1.43772 | 0.452583 |
| DUS4L | 1.374689 | 0.598577 | 3.157106 | 0.453151 |
| HADHB | 1.010933 | 0.982525 | 1.040162 | 0.454653 |
| MTMR11 | 1.128517 | 0.82198 | 1.549368 | 0.454661 |
| CDV3 | 1.007136 | 0.988456 | 1.026169 | 0.456615 |
| COL8A2 | 1.009282 | 0.985027 | 1.034135 | 0.45662 |
| GPER1 | 1.037145 | 0.942094 | 1.141786 | 0.457075 |
| KIF1A | 0.687424 | 0.255551 | 1.849146 | 0.457859 |
| PSKH1 | 0.925416 | 0.754027 | 1.135761 | 0.458239 |
| PRUNE1 | 0.96948 | 0.89311 | 1.052381 | 0.459063 |
| SLC25A30 | 0.840385 | 0.529385 | 1.33409 | 0.460823 |
| CISD1 | 0.90456 | 0.692056 | 1.182318 | 0.462849 |
| ZNF652 | 0.854332 | 0.560869 | 1.301343 | 0.463417 |
| CYC1 | 0.984435 | 0.943909 | 1.026701 | 0.464524 |
| ACACB | 0.8638 | 0.581471 | 1.283212 | 0.468413 |
| TDRD9 | 0.897756 | 0.670405 | 1.202208 | 0.469118 |
| IGFBPL1 | 0.916705 | 0.724237 | 1.160323 | 0.469499 |
| ASAP3 | 1.042429 | 0.930913 | 1.167303 | 0.47163 |
| NDUFAF4 | 0.882614 | 0.626549 | 1.243331 | 0.475093 |
| PLEKHD1 | 1.262661 | 0.665375 | 2.396112 | 0.475519 |
| FBXO8 | 1.099151 | 0.847294 | 1.425873 | 0.476473 |
| DECR1 | 0.946374 | 0.811612 | 1.103511 | 0.481902 |
| JAZF1 | 1.050698 | 0.915321 | 1.206097 | 0.482233 |
| VAPB | 0.931801 | 0.765077 | 1.134857 | 0.482522 |
| RHPN2 | 1.170053 | 0.752677 | 1.818872 | 0.485355 |
| AARS | 0.989542 | 0.960549 | 1.01941 | 0.488356 |
| PLP2 | 1.00616 | 0.988833 | 1.023792 | 0.488359 |
| SDC4 | 1.000766 | 0.998589 | 1.002948 | 0.490921 |
| RIMKLA | 0.412068 | 0.032648 | 5.20087 | 0.493121 |
| SUCLA2 | 0.953576 | 0.832184 | 1.092677 | 0.493832 |
| AIFM1 | 0.938187 | 0.78142 | 1.126406 | 0.49399 |
| SLC25A4 | 0.950867 | 0.823014 | 1.098581 | 0.494082 |
| SPX | 1.029955 | 0.946038 | 1.121317 | 0.496077 |
| ADAL | 0.80136 | 0.423469 | 1.51647 | 0.496207 |
| SLC22A31 | 0.989923 | 0.961427 | 1.019263 | 0.496735 |
| MAP1S | 0.942652 | 0.794756 | 1.11807 | 0.497611 |
| CFAP46 | 0.577437 | 0.117584 | 2.835711 | 0.498838 |
| ANO5 | 0.737246 | 0.304363 | 1.785803 | 0.499468 |
| PGAM1 | 1.070469 | 0.878314 | 1.304664 | 0.499931 |
| PPIL6 | 1.170356 | 0.739456 | 1.852354 | 0.501902 |
| SLC36A4 | 1.437905 | 0.497048 | 4.159698 | 0.502784 |
| HGD | 0.979823 | 0.922975 | 1.040172 | 0.503864 |
| VPS26B | 0.88976 | 0.630899 | 1.254834 | 0.505495 |
| DARS2 | 1.104007 | 0.824922 | 1.477511 | 0.505741 |
| FAM186B | 3.941895 | 0.06935 | 224.0591 | 0.505791 |
| CCDC126 | 0.832767 | 0.485218 | 1.429256 | 0.506675 |
| BORCS7 | 0.975881 | 0.907947 | 1.048899 | 0.507223 |
| CFAP221 | 0.381551 | 0.022018 | 6.611778 | 0.50793 |
| ALDH1L1 | 0.537814 | 0.085651 | 3.376995 | 0.508178 |
| COG2 | 1.250637 | 0.643663 | 2.429987 | 0.509295 |
| TATDN3 | 1.205128 | 0.692214 | 2.0981 | 0.509523 |
| TMEM164 | 0.943836 | 0.794685 | 1.120981 | 0.510123 |
| LRP1B | 0.701081 | 0.242409 | 2.027623 | 0.512202 |
| HERPUD1 | 0.987143 | 0.949569 | 1.026204 | 0.513392 |
| TCEA1 | 0.970258 | 0.886264 | 1.062214 | 0.513407 |
| DLAT | 1.054235 | 0.899624 | 1.235417 | 0.513943 |
| SLC4A4 | 1.025409 | 0.950708 | 1.105979 | 0.515583 |
| DYNLL2 | 1.015996 | 0.968504 | 1.065817 | 0.51588 |
| NCAM1 | 0.957063 | 0.838106 | 1.092904 | 0.516938 |
| L2HGDH | 1.234113 | 0.648051 | 2.350176 | 0.522137 |
| IBA57 | 0.509097 | 0.063097 | 4.107649 | 0.526258 |
| TNFRSF1A | 0.987117 | 0.948256 | 1.02757 | 0.526873 |
| MTIF2 | 1.092007 | 0.830632 | 1.435631 | 0.528331 |
| AP3D1 | 1.039147 | 0.921528 | 1.171779 | 0.530948 |
| PIP5KL1 | 0.846721 | 0.50266 | 1.426284 | 0.531724 |
| DHTKD1 | 1.070726 | 0.863341 | 1.327927 | 0.533845 |
| FAXDC2 | 0.955066 | 0.825855 | 1.104493 | 0.535325 |
| DUSP15 | 0.838503 | 0.47994 | 1.464949 | 0.536098 |
| RBM24 | 0.330838 | 0.009608 | 11.39194 | 0.540149 |
| IQGAP2 | 0.973274 | 0.892238 | 1.06167 | 0.541361 |
| DEPTOR | 0.989571 | 0.956762 | 1.023506 | 0.542264 |
| MKRN2 | 1.050001 | 0.89741 | 1.228539 | 0.542543 |
| HSPA9 | 1.006098 | 0.9866 | 1.02598 | 0.542622 |
| POLDIP2 | 1.012243 | 0.9733 | 1.052743 | 0.543232 |
| AACS | 0.754301 | 0.303712 | 1.87339 | 0.543528 |
| FOXE1 | 0.996518 | 0.985364 | 1.007797 | 0.543546 |
| C19orf66 | 0.936765 | 0.758677 | 1.156656 | 0.543723 |
| ATP5S | 1.213588 | 0.649648 | 2.267067 | 0.54375 |
| SLC35A4 | 0.966937 | 0.867356 | 1.07795 | 0.544295 |
| C9orf43 | 0.364915 | 0.013829 | 9.62923 | 0.546048 |
| SELENOV | 1.049268 | 0.897448 | 1.226771 | 0.546442 |
| BTBD11 | 0.957565 | 0.831322 | 1.102978 | 0.547742 |
| ANKS1B | 0.52964 | 0.066544 | 4.215516 | 0.548162 |
| ZSWIM4 | 1.100883 | 0.803321 | 1.508667 | 0.549967 |
| C1QBP | 0.972379 | 0.887053 | 1.065912 | 0.550005 |
| VTI1B | 0.940916 | 0.77025 | 1.149397 | 0.550901 |
| C4orf19 | 0.584034 | 0.099274 | 3.43592 | 0.551967 |
| AMZ2 | 0.941903 | 0.772158 | 1.148964 | 0.55496 |
| CLPB | 0.838359 | 0.466793 | 1.505689 | 0.5551 |
| WASF3 | 0.951742 | 0.807508 | 1.121738 | 0.555264 |
| ESRRG | 0.686673 | 0.196839 | 2.395452 | 0.555427 |
| SERPINA1 | 1.000442 | 0.998968 | 1.001918 | 0.557148 |
| XPNPEP3 | 0.70512 | 0.219521 | 2.264904 | 0.557316 |
| GON7 | 0.914702 | 0.678698 | 1.232772 | 0.558172 |
| LPCAT4 | 0.911642 | 0.668519 | 1.243184 | 0.558863 |
| ASB8 | 1.081604 | 0.830899 | 1.407954 | 0.55985 |
| TMEM19 | 1.083769 | 0.826641 | 1.420876 | 0.560453 |
| DLG2 | 0.801204 | 0.379557 | 1.691255 | 0.560938 |
| SUCLG1 | 0.976481 | 0.901142 | 1.058119 | 0.56127 |
| TMEM178B | 0.959546 | 0.834645 | 1.103139 | 0.56166 |
| HEBP1 | 0.981882 | 0.92283 | 1.044712 | 0.56342 |
| DLD | 1.024936 | 0.942729 | 1.11431 | 0.563674 |
| NUBPL | 1.308236 | 0.525438 | 3.257246 | 0.563745 |
| C3orf18 | 0.906528 | 0.648623 | 1.26698 | 0.565604 |
| COPS3 | 0.948121 | 0.790435 | 1.137265 | 0.565959 |
| RPS6KA6 | 0.863542 | 0.523034 | 1.425727 | 0.566303 |
| MATN2 | 0.992179 | 0.965898 | 1.019175 | 0.566469 |
| SYNE1 | 0.923026 | 0.701804 | 1.21398 | 0.566679 |
| EVA1A | 1.027159 | 0.936855 | 1.126166 | 0.568184 |
| MVP | 1.006328 | 0.984679 | 1.028452 | 0.56971 |
| PPP1R13L | 1.044678 | 0.898033 | 1.215268 | 0.571143 |
| PPID | 1.061902 | 0.862169 | 1.307907 | 0.572099 |
| CPEB4 | 1.051235 | 0.883588 | 1.250692 | 0.57296 |
| KIAA2022 | 0.254615 | 0.002108 | 30.74741 | 0.575948 |
| KCNJ1 | 0.548843 | 0.066924 | 4.501062 | 0.576295 |
| ELAC1 | 0.865382 | 0.520792 | 1.437976 | 0.576824 |
| ATP5A1 | 0.986809 | 0.941836 | 1.033931 | 0.576894 |
| MDH1 | 1.02136 | 0.948187 | 1.10018 | 0.577361 |
| KLHL8 | 0.934631 | 0.736749 | 1.185663 | 0.577565 |
| MARS2 | 1.204998 | 0.625048 | 2.323052 | 0.577661 |
| SEPHS2 | 1.01596 | 0.960814 | 1.074271 | 0.578151 |
| MRPS27 | 1.060888 | 0.861293 | 1.306737 | 0.578341 |
| NDFIP1 | 0.991019 | 0.959888 | 1.02316 | 0.579598 |
| ADCY9 | 1.03092 | 0.925009 | 1.148959 | 0.581922 |
| ID4 | 0.997769 | 0.989853 | 1.005748 | 0.582532 |
| MINDY1 | 1.019221 | 0.952307 | 1.090838 | 0.582659 |
| DNAJB9 | 0.983082 | 0.924932 | 1.044887 | 0.583357 |
| GNS | 1.010327 | 0.973906 | 1.048109 | 0.583377 |
| PVALB | 0.882509 | 0.564062 | 1.38074 | 0.584182 |
| PRR15L | 0.994258 | 0.973776 | 1.01517 | 0.587652 |
| TMEM38A | 0.891269 | 0.587464 | 1.352186 | 0.588337 |
| TMEM14B | 0.969762 | 0.866824 | 1.084924 | 0.591751 |
| LGMN | 0.992331 | 0.964614 | 1.020845 | 0.594305 |
| TTC30B | 0.880089 | 0.549597 | 1.409318 | 0.594926 |
| SLC25A5 | 0.996494 | 0.983674 | 1.009481 | 0.594992 |
| TFB2M | 1.053696 | 0.868304 | 1.278671 | 0.596292 |
| HIGD1A | 0.982945 | 0.922147 | 1.047751 | 0.597459 |
| CMTM4 | 0.987568 | 0.942651 | 1.034625 | 0.598382 |
| PCCB | 1.044363 | 0.888585 | 1.227451 | 0.59841 |
| MT3 | 0.783038 | 0.314539 | 1.949355 | 0.599188 |
| UQCRFS1 | 0.963149 | 0.836729 | 1.10867 | 0.600975 |
| SH3GL1 | 0.98022 | 0.909489 | 1.056452 | 0.601096 |
| TMEM43 | 1.006764 | 0.981603 | 1.032569 | 0.601653 |
| ZNF275 | 0.909316 | 0.635164 | 1.3018 | 0.603574 |
| TP53INP2 | 0.979659 | 0.906434 | 1.0588 | 0.604125 |
| MLYCD | 0.657121 | 0.13365 | 3.230881 | 0.605345 |
| MCCC2 | 1.016372 | 0.955328 | 1.081317 | 0.607342 |
| DEFB1 | 0.966781 | 0.849031 | 1.100862 | 0.610178 |
| YBX3 | 0.981226 | 0.912147 | 1.055535 | 0.610853 |
| COQ3 | 1.125501 | 0.713718 | 1.774863 | 0.610944 |
| NIPSNAP2 | 1.016709 | 0.953709 | 1.083871 | 0.611638 |
| PRSS22 | 1.037329 | 0.899515 | 1.196256 | 0.614328 |
| PSAT1 | 0.986133 | 0.933592 | 1.041632 | 0.617179 |
| LRRC2 | 0.965423 | 0.840293 | 1.109187 | 0.619306 |
| CXorf40A | 0.831441 | 0.399336 | 1.731108 | 0.621767 |
| KCNA2 | 0.548383 | 0.049557 | 6.068178 | 0.624243 |
| PRKCQ | 0.959824 | 0.814473 | 1.131113 | 0.624531 |
| KIAA0141 | 0.94057 | 0.735916 | 1.202137 | 0.624555 |
| GUF1 | 1.101031 | 0.748705 | 1.619155 | 0.62474 |
| TM6SF1 | 0.915132 | 0.640917 | 1.30667 | 0.625526 |
| OAT | 0.981333 | 0.909711 | 1.058592 | 0.62601 |
| PGK1 | 0.99051 | 0.953236 | 1.029241 | 0.626079 |
| SNTA1 | 0.995865 | 0.979326 | 1.012683 | 0.627715 |
| OSBPL1A | 0.93046 | 0.694481 | 1.246624 | 0.629141 |
| PPP1R13B | 0.962857 | 0.825625 | 1.122898 | 0.629473 |
| GPD1L | 1.012746 | 0.961873 | 1.06631 | 0.630049 |
| DEFB132 | 1.50E-21 | 2.09E-106 | 1.08E+64 | 0.630531 |
| MAIP1 | 1.07927 | 0.790347 | 1.473813 | 0.631312 |
| ACSL6 | 0.221234 | 0.00045 | 108.7247 | 0.6333 |
| LYRM7 | 0.907402 | 0.608736 | 1.352604 | 0.633309 |
| TBCE | 1.032891 | 0.903664 | 1.180598 | 0.635111 |
| INHBC | 0.52592 | 0.036891 | 7.497454 | 0.635503 |
| PRMT3 | 0.916999 | 0.640753 | 1.312343 | 0.635666 |
| FAM131C | 1.428873 | 0.324985 | 6.282374 | 0.636677 |
| ABCC4 | 0.921377 | 0.654377 | 1.297319 | 0.639052 |
| STXBP5L | 0.866595 | 0.474802 | 1.581684 | 0.640912 |
| ATP6V1E2 | 0.708407 | 0.166201 | 3.019481 | 0.641189 |
| FICD | 0.9021 | 0.583581 | 1.394468 | 0.642906 |
| FITM2 | 0.938641 | 0.717859 | 1.227326 | 0.643494 |
| LPAR5 | 1.010709 | 0.965697 | 1.05782 | 0.64675 |
| PRKAG1 | 1.05454 | 0.840075 | 1.323755 | 0.647117 |
| BCKDHB | 0.929215 | 0.678322 | 1.272907 | 0.647523 |
| TG | 0.999954 | 0.999757 | 1.000151 | 0.648477 |
| KIF3B | 1.027979 | 0.912773 | 1.157726 | 0.649095 |
| WDR45B | 1.026421 | 0.917172 | 1.148682 | 0.649704 |
| MTCH2 | 1.017975 | 0.942147 | 1.099905 | 0.651938 |
| NDUFS4 | 0.985499 | 0.924878 | 1.050094 | 0.652028 |
| PLA2G12A | 0.948635 | 0.75308 | 1.194969 | 0.654372 |
| UBE2D4 | 1.08072 | 0.76851 | 1.519766 | 0.655402 |
| STK40 | 0.971405 | 0.854717 | 1.104023 | 0.656803 |
| HOXA13 | 4.425809 | 0.006255 | 3131.392 | 0.656831 |
| VDAC2 | 0.972237 | 0.858305 | 1.101293 | 0.657952 |
| TRIB3 | 0.993958 | 0.967622 | 1.021011 | 0.658261 |
| ETFDH | 0.922501 | 0.644654 | 1.320101 | 0.659092 |
| ZNF330 | 1.025718 | 0.915495 | 1.149212 | 0.661535 |
| AAGAB | 0.964996 | 0.822262 | 1.132506 | 0.662616 |
| LRIG1 | 0.960492 | 0.801367 | 1.151213 | 0.662697 |
| BAP1 | 1.038426 | 0.876493 | 1.230277 | 0.662895 |
| TPO | 0.999597 | 0.997786 | 1.001411 | 0.663006 |
| TTC7B | 1.208787 | 0.514946 | 2.837513 | 0.663178 |
| MPPED2 | 0.952402 | 0.764709 | 1.186163 | 0.663217 |
| ATP6V1H | 0.962491 | 0.810135 | 1.1435 | 0.66369 |
| TTC30A | 0.899215 | 0.555403 | 1.455855 | 0.665644 |
| CEBPG | 0.978657 | 0.886984 | 1.079804 | 0.667254 |
| PTPN4 | 1.03725 | 0.877284 | 1.226386 | 0.668682 |
| SYT9 | 1.419614 | 0.284291 | 7.088877 | 0.669349 |
| TIMM8A | 0.813436 | 0.314072 | 2.10677 | 0.670637 |
| HADHA | 0.991473 | 0.952921 | 1.031586 | 0.672159 |
| RNF150 | 0.890523 | 0.520145 | 1.524635 | 0.672563 |
| GFM1 | 1.066792 | 0.790302 | 1.440013 | 0.67272 |
| GOT2 | 0.986118 | 0.924011 | 1.052401 | 0.673634 |
| GCSH | 0.961031 | 0.797753 | 1.157727 | 0.675667 |
| ATP5F1 | 0.986132 | 0.923361 | 1.05317 | 0.677283 |
| EML1 | 1.067684 | 0.783479 | 1.454984 | 0.678336 |
| TMC6 | 1.017934 | 0.935696 | 1.1074 | 0.679191 |
| DIO2 | 0.997243 | 0.984259 | 1.010398 | 0.67967 |
| EIF2AK3 | 0.958562 | 0.783904 | 1.172134 | 0.680063 |
| FAM114A2 | 0.851931 | 0.397572 | 1.825547 | 0.680257 |
| CS | 1.020033 | 0.928146 | 1.121017 | 0.680469 |
| GALK2 | 1.216849 | 0.476586 | 3.10694 | 0.681532 |
| CCDC149 | 0.856051 | 0.40664 | 1.802142 | 0.682374 |
| KIAA1614 | 0.799382 | 0.27238 | 2.346029 | 0.683547 |
| MLH1 | 1.049191 | 0.832844 | 1.321738 | 0.683602 |
| MRS2 | 0.922761 | 0.625419 | 1.361468 | 0.685426 |
| RTN4IP1 | 0.922683 | 0.6251 | 1.361932 | 0.685437 |
| FAM167A | 0.994923 | 0.970494 | 1.019968 | 0.688227 |
| ELMO1 | 0.982117 | 0.899247 | 1.072623 | 0.688261 |
| COX10 | 0.890861 | 0.506078 | 1.568204 | 0.688756 |
| ARHGEF28 | 1.060912 | 0.7923 | 1.420591 | 0.691397 |
| ASNS | 0.974677 | 0.858552 | 1.106509 | 0.691899 |
| FAM151A | 0.659308 | 0.082827 | 5.248113 | 0.693893 |
| GOT1 | 0.991878 | 0.952092 | 1.033327 | 0.696227 |
| CFL2 | 1.056197 | 0.802319 | 1.39041 | 0.696695 |
| ADH5 | 0.979015 | 0.879482 | 1.089812 | 0.698235 |
| CLN5 | 0.97237 | 0.842881 | 1.121753 | 0.700783 |
| SLC6A17 | 1.190023 | 0.489093 | 2.895473 | 0.701365 |
| GOSR2 | 0.87251 | 0.432584 | 1.759829 | 0.70321 |
| MAPK4 | 0.915301 | 0.579876 | 1.44475 | 0.703922 |
| LGALS3 | 1.001004 | 0.995818 | 1.006217 | 0.704928 |
| USP2 | 1.073069 | 0.744656 | 1.546322 | 0.70519 |
| SYT13 | 0.970246 | 0.828701 | 1.135966 | 0.707334 |
| GATB | 1.283939 | 0.347044 | 4.750117 | 0.708075 |
| IDH3A | 0.903618 | 0.527852 | 1.546882 | 0.711755 |
| TMEM173 | 0.993633 | 0.960397 | 1.02802 | 0.712909 |
| EHHADH | 1.090927 | 0.683948 | 1.740077 | 0.714868 |
| XPOT | 0.989348 | 0.933273 | 1.048792 | 0.719049 |
| BPHL | 1.196833 | 0.449501 | 3.186667 | 0.719139 |
| CA5A | 0.117385 | 9.97E-07 | 13819.91 | 0.719142 |
| HRH2 | 0.57911 | 0.029327 | 11.43535 | 0.719653 |
| CEP70 | 1.041721 | 0.83294 | 1.302835 | 0.720214 |
| UNG | 0.980956 | 0.882976 | 1.089808 | 0.720244 |
| TMEM38B | 1.018576 | 0.920139 | 1.127543 | 0.722641 |
| CLUH | 0.954078 | 0.735417 | 1.237753 | 0.72337 |
| AIMP2 | 1.019986 | 0.913955 | 1.138319 | 0.723818 |
| TRIM37 | 1.053596 | 0.787541 | 1.409533 | 0.725149 |
| PRDX3 | 1.007249 | 0.967491 | 1.04864 | 0.725197 |
| ACOT11 | 0.901932 | 0.506271 | 1.606812 | 0.726098 |
| PRPS1 | 0.988169 | 0.924397 | 1.056339 | 0.72658 |
| ETFBKMT | 0.725904 | 0.120253 | 4.381877 | 0.726917 |
| TUB | 0.955481 | 0.739533 | 1.234488 | 0.727545 |
| PPP2R2B | 1.365345 | 0.235238 | 7.924604 | 0.728538 |
| UBE2I | 1.028019 | 0.878614 | 1.202829 | 0.730186 |
| ATP7B | 0.732834 | 0.124793 | 4.303476 | 0.730736 |
| TFCP2L1 | 0.982149 | 0.885819 | 1.088956 | 0.732368 |
| SLC39A9 | 0.967649 | 0.801343 | 1.168469 | 0.732506 |
| DIO1 | 0.998761 | 0.991686 | 1.005887 | 0.732557 |
| UCHL5 | 0.898712 | 0.483766 | 1.669573 | 0.735405 |
| SLC1A1 | 0.98903 | 0.927734 | 1.054376 | 0.735433 |
| EHD2 | 0.993461 | 0.956307 | 1.032058 | 0.735848 |
| RAD51D | 0.805142 | 0.226014 | 2.868205 | 0.738098 |
| EPM2A | 1.208596 | 0.398036 | 3.669784 | 0.738129 |
| EIF2B2 | 0.977635 | 0.856198 | 1.116296 | 0.738197 |
| COQ5 | 0.970672 | 0.81507 | 1.155981 | 0.73844 |
| ATP5B | 0.998861 | 0.992179 | 1.005587 | 0.73923 |
| SLC16A7 | 0.609242 | 0.032832 | 11.30516 | 0.739492 |
| MAGI3 | 1.02267 | 0.896087 | 1.167135 | 0.739499 |
| PRKAA2 | 1.114173 | 0.588615 | 2.108987 | 0.739832 |
| FAM20C | 1.009523 | 0.95453 | 1.067685 | 0.74016 |
| ACSF2 | 0.990248 | 0.934349 | 1.049492 | 0.740981 |
| CMC2 | 1.254564 | 0.326326 | 4.823189 | 0.741343 |
| GATM | 1.013474 | 0.934862 | 1.098696 | 0.745263 |
| RIC8B | 0.938123 | 0.637999 | 1.37943 | 0.7454 |
| ALDOA | 1.00192 | 0.990377 | 1.013597 | 0.745631 |
| NMNAT3 | 0.890839 | 0.442794 | 1.792241 | 0.745872 |
| PDLIM4 | 1.002466 | 0.987632 | 1.017523 | 0.746106 |
| SYNPR | 1.036365 | 0.834194 | 1.287532 | 0.746994 |
| IVD | 0.992127 | 0.945351 | 1.041217 | 0.748377 |
| SLC25A33 | 0.905817 | 0.494546 | 1.659107 | 0.748703 |
| MED15 | 0.970415 | 0.807315 | 1.166466 | 0.749065 |
| SDHC | 1.051268 | 0.773843 | 1.428151 | 0.749092 |
| GPHN | 1.032954 | 0.846194 | 1.260933 | 0.749999 |
| SLC39A11 | 1.012348 | 0.93856 | 1.091939 | 0.750611 |
| KIT | 1.005869 | 0.970215 | 1.042833 | 0.750646 |
| ST3GAL4 | 0.96798 | 0.791057 | 1.184471 | 0.751988 |
| PDE9A | 1.022149 | 0.892285 | 1.170913 | 0.752004 |
| C17orf75 | 0.911673 | 0.512857 | 1.620624 | 0.752721 |
| NT5M | 0.894928 | 0.448389 | 1.786162 | 0.752884 |
| FASTKD5 | 1.054239 | 0.758422 | 1.465438 | 0.753261 |
| ZFYVE9 | 0.969497 | 0.79819 | 1.17757 | 0.754836 |
| WDR72 | 0.984548 | 0.892807 | 1.085716 | 0.755003 |
| HMGCR | 1.021761 | 0.892301 | 1.170003 | 0.755472 |
| DIXDC1 | 0.946009 | 0.666382 | 1.342972 | 0.756204 |
| PXMP2 | 0.958012 | 0.730292 | 1.256741 | 0.756749 |
| C11orf80 | 1.039211 | 0.814408 | 1.326066 | 0.757126 |
| TPI1 | 0.996984 | 0.978054 | 1.01628 | 0.757451 |
| ANKRD46 | 0.945234 | 0.660117 | 1.3535 | 0.758479 |
| FKBP4 | 1.005682 | 0.969404 | 1.043317 | 0.762464 |
| BRI3BP | 0.915182 | 0.513776 | 1.630203 | 0.763497 |
| ATRN | 1.013336 | 0.929511 | 1.10472 | 0.76363 |
| LCA5L | 0.792063 | 0.172702 | 3.632633 | 0.764191 |
| CCDC51 | 0.950432 | 0.681751 | 1.324999 | 0.764252 |
| IMMT | 1.016012 | 0.915649 | 1.127376 | 0.764679 |
| COMMD8 | 0.96996 | 0.793345 | 1.185893 | 0.766147 |
| DGKI | 1.023022 | 0.879416 | 1.190078 | 0.768048 |
| CNPPD1 | 0.991389 | 0.935281 | 1.050863 | 0.771094 |
| AS3MT | 1.917101 | 0.023168 | 158.635 | 0.772684 |
| PFKFB2 | 0.989886 | 0.923905 | 1.060579 | 0.772708 |
| DNAJA2 | 1.025458 | 0.86444 | 1.216468 | 0.772995 |
| ALDH1B1 | 0.98438 | 0.884369 | 1.095701 | 0.773343 |
| BBS4 | 0.964867 | 0.755976 | 1.231478 | 0.773874 |
| LRRFIP2 | 0.932636 | 0.579336 | 1.501392 | 0.774052 |
| ENPP1 | 0.992325 | 0.941241 | 1.046181 | 0.775084 |
| MARVELD2 | 0.986369 | 0.897371 | 1.084194 | 0.776048 |
| ALDH7A1 | 1.014862 | 0.916572 | 1.123691 | 0.776534 |
| LY6E | 0.998784 | 0.99041 | 1.007229 | 0.777068 |
| FAM81A | 0.894531 | 0.41162 | 1.943994 | 0.778378 |
| S100A11 | 0.999724 | 0.9978 | 1.001651 | 0.778394 |
| FBXO36 | 1.063653 | 0.691937 | 1.635059 | 0.778484 |
| NCR3LG1 | 0.801031 | 0.169701 | 3.781065 | 0.779326 |
| TACC2 | 0.963982 | 0.745485 | 1.24652 | 0.779699 |
| LIFR | 0.983299 | 0.873221 | 1.107253 | 0.780983 |
| PAICS | 1.028759 | 0.842229 | 1.2566 | 0.78118 |
| YKT6 | 1.014548 | 0.915944 | 1.123768 | 0.781874 |
| WDR17 | 1.155438 | 0.409874 | 3.257191 | 0.784674 |
| PHKB | 0.975722 | 0.817528 | 1.164528 | 0.785381 |
| SRPRA | 0.992303 | 0.938601 | 1.049078 | 0.785481 |
| AAK1 | 0.961048 | 0.721814 | 1.279572 | 0.785597 |
| KLF16 | 0.969224 | 0.77362 | 1.214285 | 0.785775 |
| SPATA13 | 1.041577 | 0.776328 | 1.397456 | 0.785894 |
| RNF187 | 0.998056 | 0.984139 | 1.01217 | 0.785957 |
| MRPL42 | 1.149171 | 0.420387 | 3.141379 | 0.786397 |
| PIGX | 1.063705 | 0.678712 | 1.667082 | 0.787625 |
| ACSS2 | 1.045513 | 0.75526 | 1.447312 | 0.788512 |
| DBT | 0.933412 | 0.563876 | 1.545125 | 0.788726 |
| POLR3B | 1.082149 | 0.606973 | 1.929324 | 0.789 |
| NIM1K | 0.861801 | 0.287615 | 2.582274 | 0.790522 |
| ETFRF1 | 0.9751 | 0.808141 | 1.176552 | 0.792431 |
| ZDHHC16 | 0.97161 | 0.783989 | 1.204133 | 0.792485 |
| NR0B2 | 0.756522 | 0.093503 | 6.120902 | 0.793652 |
| MRPS22 | 0.924029 | 0.511046 | 1.670748 | 0.793735 |
| RYR2 | 1.078064 | 0.609729 | 1.906128 | 0.796016 |
| ATP6V1D | 0.98957 | 0.913877 | 1.071533 | 0.796227 |
| BID | 1.016889 | 0.895348 | 1.154929 | 0.796503 |
| DIRC2 | 0.950428 | 0.643868 | 1.402948 | 0.798031 |
| ACADM | 0.983766 | 0.867772 | 1.115264 | 0.798182 |
| ACSL1 | 0.988684 | 0.906079 | 1.078819 | 0.798214 |
| C16orf96 | 1.801482 | 0.019731 | 164.4751 | 0.798288 |
| WDR31 | 1.065178 | 0.655642 | 1.730522 | 0.798709 |
| HSDL1 | 0.97507 | 0.803007 | 1.184001 | 0.798824 |
| COPS7A | 0.984409 | 0.872264 | 1.110972 | 0.798998 |
| CCDC151 | 0.94202 | 0.593736 | 1.494607 | 0.799795 |
| PPCS | 1.013354 | 0.914559 | 1.122821 | 0.799907 |
| ATPAF1 | 1.022485 | 0.860449 | 1.215036 | 0.800581 |
| DCTPP1 | 0.984577 | 0.872587 | 1.11094 | 0.800813 |
| PUS7 | 0.948029 | 0.624072 | 1.440153 | 0.80245 |
| LGI3 | 1.011178 | 0.926378 | 1.103741 | 0.803556 |
| CRAT | 0.978022 | 0.82007 | 1.166397 | 0.804692 |
| GATA5 | 1.021637 | 0.862067 | 1.210745 | 0.804869 |
| ADGRA3 | 0.974779 | 0.795402 | 1.19461 | 0.805536 |
| SLC7A1 | 1.016885 | 0.888597 | 1.163693 | 0.807733 |
| ALDH6A1 | 1.015337 | 0.89808 | 1.147904 | 0.80793 |
| ANKEF1 | 1.04188 | 0.746365 | 1.454401 | 0.809507 |
| HTATIP2 | 0.989373 | 0.905508 | 1.081006 | 0.81312 |
| OGDH | 1.003499 | 0.974738 | 1.033108 | 0.813879 |
| L3MBTL4 | 1.106926 | 0.470771 | 2.602725 | 0.815853 |
| TAGLN2 | 1.000927 | 0.993127 | 1.008787 | 0.816493 |
| CYP17A1 | 0.926748 | 0.4866 | 1.765025 | 0.816973 |
| HADH | 1.010235 | 0.925847 | 1.102315 | 0.81902 |
| MPP6 | 0.917919 | 0.440412 | 1.913154 | 0.819202 |
| NARS | 1.005453 | 0.95927 | 1.053859 | 0.820681 |
| RMDN1 | 0.975286 | 0.784811 | 1.211991 | 0.821419 |
| PLEKHN1 | 1.03002 | 0.796413 | 1.332151 | 0.821679 |
| COQ9 | 0.987667 | 0.886547 | 1.10032 | 0.821828 |
| GPR155 | 1.049221 | 0.690322 | 1.594712 | 0.822022 |
| SLC26A7 | 0.998453 | 0.985041 | 1.012047 | 0.822417 |
| NT5DC1 | 0.974826 | 0.77993 | 1.218424 | 0.822733 |
| TMEM135 | 1.036462 | 0.756577 | 1.419887 | 0.823535 |
| BCAP29 | 1.009919 | 0.92592 | 1.101538 | 0.823713 |
| LSR | 0.997094 | 0.971814 | 1.023031 | 0.82421 |
| TMCO4 | 1.017486 | 0.872823 | 1.186126 | 0.824664 |
| LARS2 | 1.046719 | 0.698491 | 1.568555 | 0.824899 |
| ST7 | 0.962528 | 0.685273 | 1.351957 | 0.825615 |
| CBLB | 0.942975 | 0.559121 | 1.590356 | 0.825733 |
| TRMT10A | 1.111974 | 0.43104 | 2.868614 | 0.826256 |
| AFG1L | 1.430183 | 0.058472 | 34.9811 | 0.826373 |
| SLC31A1 | 0.972284 | 0.753755 | 1.25417 | 0.828684 |
| NMNAT1 | 0.952573 | 0.611462 | 1.483979 | 0.829911 |
| PDHA1 | 1.009553 | 0.925621 | 1.101095 | 0.830011 |
| ATP5C1 | 0.996389 | 0.963925 | 1.029947 | 0.830523 |
| ERMP1 | 1.005495 | 0.955641 | 1.057951 | 0.832718 |
| CYCS | 0.993314 | 0.933331 | 1.057153 | 0.832821 |
| PFKM | 0.986451 | 0.868912 | 1.119891 | 0.833096 |
| RAP1GAP | 0.998566 | 0.985187 | 1.012127 | 0.834861 |
| PNPO | 1.011809 | 0.906074 | 1.129882 | 0.834865 |
| ISCA1 | 0.981215 | 0.820365 | 1.173602 | 0.835542 |
| IARS | 0.993757 | 0.935829 | 1.055271 | 0.838066 |
| SLC25A3 | 1.006671 | 0.944405 | 1.073043 | 0.838268 |
| MYO10 | 0.985748 | 0.858437 | 1.13194 | 0.838784 |
| WWOX | 1.028739 | 0.782663 | 1.352184 | 0.839031 |
| PAFAH2 | 1.014671 | 0.878812 | 1.171533 | 0.842591 |
| OXCT1 | 1.010838 | 0.90861 | 1.124569 | 0.842915 |
| PRPSAP1 | 1.017201 | 0.857769 | 1.206266 | 0.84455 |
| VDAC3 | 0.993393 | 0.929395 | 1.061799 | 0.845324 |
| SLC39A14 | 1.003691 | 0.967086 | 1.041681 | 0.845912 |
| PPA2 | 0.984956 | 0.842989 | 1.150832 | 0.84862 |
| CHAC1 | 0.968684 | 0.693321 | 1.353412 | 0.852088 |
| KLK4 | 0.996999 | 0.965619 | 1.029399 | 0.853847 |
| AK6 | 1.023614 | 0.797385 | 1.314026 | 0.854676 |
| TRIM2 | 0.993639 | 0.926626 | 1.065499 | 0.857851 |
| FECH | 1.021343 | 0.807432 | 1.291924 | 0.8602 |
| MMP15 | 0.998307 | 0.979462 | 1.017514 | 0.86164 |
| SLC30A9 | 1.015592 | 0.853288 | 1.208768 | 0.861753 |
| BCAN | 0.621817 | 0.002946 | 131.2455 | 0.861878 |
| RIOX2 | 0.98895 | 0.871268 | 1.122528 | 0.863522 |
| AFG3L2 | 1.013591 | 0.868335 | 1.183146 | 0.864175 |
| PDE11A | 1.214594 | 0.129572 | 11.38546 | 0.864803 |
| KLHL3 | 1.02073 | 0.805012 | 1.294254 | 0.865495 |
| COA1 | 1.067328 | 0.500459 | 2.27629 | 0.866098 |
| TDGF1 | 1.343566 | 0.038383 | 47.03045 | 0.870676 |
| EEF1AKMT3 | 0.97491 | 0.717206 | 1.325211 | 0.871121 |
| CLCN5 | 1.09731 | 0.357334 | 3.369643 | 0.87113 |
| C16orf46 | 1.037001 | 0.665955 | 1.614781 | 0.872253 |
| WSB2 | 0.993589 | 0.918452 | 1.074872 | 0.872628 |
| CD164 | 0.998349 | 0.978327 | 1.018781 | 0.872978 |
| CTH | 1.035698 | 0.672504 | 1.595038 | 0.873511 |
| PPM1H | 0.97058 | 0.66659 | 1.4132 | 0.876211 |
| UQCRC2 | 1.005342 | 0.939282 | 1.076048 | 0.877901 |
| SORBS2 | 0.995779 | 0.943394 | 1.051073 | 0.878075 |
| VWA8 | 1.033743 | 0.673281 | 1.587189 | 0.879426 |
| PANK1 | 0.967962 | 0.632376 | 1.481634 | 0.880829 |
| FTCDNL1 | 0.95342 | 0.508109 | 1.789006 | 0.881911 |
| CLCN4 | 0.979697 | 0.744271 | 1.289592 | 0.883702 |
| PAIP2B | 0.98265 | 0.77679 | 1.243067 | 0.883986 |
| LONP1 | 0.995178 | 0.930188 | 1.064708 | 0.888428 |
| HSPD1 | 1.002172 | 0.971938 | 1.033347 | 0.889597 |
| SCO1 | 1.039212 | 0.603136 | 1.790578 | 0.8898 |
| SYNJ2BP | 0.98078 | 0.742153 | 1.296133 | 0.891477 |
| HSPA4L | 1.060584 | 0.454872 | 2.47287 | 0.891678 |
| DHX29 | 1.010757 | 0.866294 | 1.17931 | 0.891844 |
| CYTH3 | 0.991174 | 0.870451 | 1.128641 | 0.893577 |
| PSPH | 1.012348 | 0.845697 | 1.21184 | 0.893613 |
| CDON | 0.989724 | 0.850264 | 1.152057 | 0.893954 |
| ACSS1 | 1.011523 | 0.853334 | 1.199036 | 0.894952 |
| BDH1 | 0.979373 | 0.712365 | 1.34646 | 0.897885 |
| NETO2 | 0.960586 | 0.503902 | 1.831161 | 0.902772 |
| MRPL19 | 1.018926 | 0.751522 | 1.381476 | 0.903915 |
| NUP62CL | 0.974404 | 0.638517 | 1.486982 | 0.904297 |
| CDC42EP1 | 1.001803 | 0.972804 | 1.031666 | 0.904336 |
| WIPI1 | 1.017418 | 0.766474 | 1.350521 | 0.904879 |
| TARS | 1.006499 | 0.904305 | 1.120242 | 0.905602 |
| COPS4 | 0.988992 | 0.82277 | 1.188796 | 0.906149 |
| CLCNKA | 0.990796 | 0.848222 | 1.157333 | 0.907134 |
| NANP | 1.06248 | 0.37789 | 2.987283 | 0.908519 |
| NPY4R | 1.644965 | 0.000298 | 9071.299 | 0.909847 |
| C14orf159 | 0.988882 | 0.813753 | 1.2017 | 0.910487 |
| GHITM | 1.001366 | 0.977736 | 1.025567 | 0.910792 |
| FBXL17 | 1.012957 | 0.801902 | 1.279561 | 0.914 |
| SLC35E3 | 0.856523 | 0.051043 | 14.37285 | 0.914287 |
| EPHB1 | 0.983909 | 0.731774 | 1.322917 | 0.914478 |
| PCMTD1 | 1.006725 | 0.887244 | 1.142296 | 0.917182 |
| PEG3 | 0.977525 | 0.636859 | 1.50042 | 0.917186 |
| FAM151B | 0.78055 | 0.007197 | 84.64941 | 0.917471 |
| CIAPIN1 | 0.988035 | 0.785448 | 1.242875 | 0.918109 |
| RARG | 0.994235 | 0.889934 | 1.11076 | 0.918558 |
| ANXA1 | 1.000167 | 0.996898 | 1.003447 | 0.920358 |
| ADGRV1 | 1.052891 | 0.374858 | 2.957329 | 0.92208 |
| GDPD1 | 1.024507 | 0.627661 | 1.672263 | 0.922845 |
| SPR | 1.002839 | 0.946731 | 1.062272 | 0.923123 |
| PRKN | 0.918128 | 0.161094 | 5.232697 | 0.923363 |
| PCBD2 | 1.053008 | 0.367465 | 3.017497 | 0.923395 |
| NFE2L1 | 0.998718 | 0.972207 | 1.025953 | 0.925569 |
| TMEM232 | 1.16345 | 0.048348 | 27.99731 | 0.925676 |
| TMEM116 | 0.970188 | 0.505815 | 1.860886 | 0.927433 |
| C5orf30 | 0.98837 | 0.764191 | 1.278312 | 0.928976 |
| FH | 0.996752 | 0.926013 | 1.072893 | 0.930963 |
| AGL | 0.985975 | 0.711216 | 1.36688 | 0.932464 |
| TANGO2 | 1.007847 | 0.840281 | 1.208828 | 0.93286 |
| ADIPOR2 | 0.996729 | 0.922096 | 1.077402 | 0.934233 |
| HCN2 | 0.960281 | 0.363755 | 2.535056 | 0.934782 |
| PRKD2 | 1.004537 | 0.901013 | 1.119955 | 0.934991 |
| EARS2 | 0.984933 | 0.682181 | 1.422046 | 0.935428 |
| ARHGAP24 | 1.006013 | 0.865641 | 1.169148 | 0.937681 |
| ACO2 | 0.998055 | 0.949158 | 1.049472 | 0.939459 |
| ZNF480 | 0.979589 | 0.574853 | 1.669288 | 0.939555 |
| VASN | 0.999286 | 0.980933 | 1.017982 | 0.939807 |
| GSTK1 | 0.998449 | 0.957741 | 1.040888 | 0.941747 |
| TRIP4 | 0.992026 | 0.79807 | 1.23312 | 0.942504 |
| MOCS2 | 1.007687 | 0.817588 | 1.241986 | 0.942769 |
| DIP2C | 0.986049 | 0.670666 | 1.449741 | 0.943047 |
| MRPS10 | 0.994906 | 0.860907 | 1.14976 | 0.944831 |
| BMP1 | 0.996273 | 0.893857 | 1.110425 | 0.946216 |
| ERCC8 | 0.949642 | 0.201355 | 4.47876 | 0.94794 |
| ACAT1 | 0.997288 | 0.91792 | 1.083519 | 0.948823 |
| PPIF | 1.00113 | 0.965926 | 1.037616 | 0.950712 |
| NUDT7 | 0.98765 | 0.659621 | 1.478808 | 0.951887 |
| SLC9A3R1 | 1.001154 | 0.962006 | 1.041895 | 0.954801 |
| PTCD2 | 0.962817 | 0.255637 | 3.626301 | 0.955339 |
| FA2H | 0.992614 | 0.75525 | 1.304579 | 0.957602 |
| DTWD2 | 0.988427 | 0.632686 | 1.544191 | 0.959216 |
| COMMD10 | 1.005419 | 0.815428 | 1.239676 | 0.959668 |
| SH3BP5 | 0.992752 | 0.745837 | 1.321411 | 0.960239 |
| MAN1C1 | 0.996952 | 0.882399 | 1.126377 | 0.960905 |
| ZBTB2 | 1.002006 | 0.92227 | 1.088635 | 0.962229 |
| NSF | 1.00658 | 0.765509 | 1.323567 | 0.962552 |
| SH2D3A | 1.007254 | 0.743036 | 1.365427 | 0.962861 |
| HARBI1 | 1.033666 | 0.230436 | 4.636715 | 0.96551 |
| RTKN | 1.003902 | 0.836952 | 1.204154 | 0.966524 |
| ALDH2 | 1.001354 | 0.937198 | 1.069901 | 0.968056 |
| ALDH1A1 | 0.999779 | 0.988882 | 1.010796 | 0.968468 |
| SLC26A4 | 1.000191 | 0.990226 | 1.010255 | 0.9702 |
| SNX19 | 0.997114 | 0.855464 | 1.16222 | 0.970513 |
| SNN | 0.998712 | 0.929766 | 1.072771 | 0.971829 |
| DAND5 | 1.040142 | 0.115943 | 9.331231 | 0.971953 |
| LDHD | 1.001203 | 0.933865 | 1.073398 | 0.972991 |
| SAR1B | 0.993366 | 0.672958 | 1.466325 | 0.973275 |
| RPP14 | 1.014006 | 0.419276 | 2.452346 | 0.975374 |
| METTL7A | 0.999855 | 0.990027 | 1.009781 | 0.977046 |
| SYTL3 | 1.001282 | 0.914432 | 1.096382 | 0.977914 |
| MSANTD4 | 0.993296 | 0.5996 | 1.645494 | 0.979163 |
| SEC23B | 1.000891 | 0.934644 | 1.071833 | 0.979672 |
| NADK2 | 0.99588 | 0.719122 | 1.37915 | 0.980174 |
| STARD7 | 0.9994 | 0.950622 | 1.050681 | 0.981241 |
| HSD17B4 | 0.999402 | 0.946483 | 1.055281 | 0.982819 |
| PDHB | 1.002706 | 0.782663 | 1.284614 | 0.982942 |
| ZDHHC23 | 1.008082 | 0.458248 | 2.21764 | 0.984034 |
| TNFRSF12A | 1.000081 | 0.991979 | 1.008249 | 0.984403 |
| CCNC | 0.998145 | 0.817535 | 1.218657 | 0.985458 |
| CPEB3 | 0.994836 | 0.558809 | 1.771085 | 0.985964 |
| MYL12A | 1.00021 | 0.976994 | 1.023977 | 0.986048 |
| COX15 | 0.997783 | 0.777846 | 1.279907 | 0.986062 |
| SLC38A1 | 0.999408 | 0.933694 | 1.069746 | 0.986376 |
| SPATA4 | 0.980451 | 0.090116 | 10.66717 | 0.987066 |
| NNT | 1.000641 | 0.918192 | 1.090494 | 0.988341 |
| MXRA8 | 1.000067 | 0.990878 | 1.009342 | 0.988573 |
| SCP2 | 1.000942 | 0.87512 | 1.144854 | 0.989038 |
| CLIC2 | 0.99954 | 0.932249 | 1.071689 | 0.98968 |
| TIFA | 1.001724 | 0.76667 | 1.308843 | 0.989929 |
| NDUFA5 | 1.000733 | 0.875327 | 1.144106 | 0.991439 |
| PLLP | 1.003133 | 0.553649 | 1.817535 | 0.991769 |
| MRPS33 | 1.000659 | 0.855753 | 1.170103 | 0.993411 |
| UGT8 | 0.999065 | 0.789047 | 1.264982 | 0.993802 |
| ACO1 | 1.000177 | 0.952297 | 1.050464 | 0.994347 |
| ACADSB | 0.999213 | 0.797158 | 1.252483 | 0.994548 |
| FAM234B | 0.999165 | 0.785625 | 1.270747 | 0.994568 |
| TAF4B | 1.002688 | 0.430792 | 2.333803 | 0.99503 |
| AP5M1 | 0.998429 | 0.570323 | 1.747887 | 0.995608 |
| RNF14 | 0.999609 | 0.841666 | 1.187191 | 0.996442 |
| PNPLA4 | 1.000079 | 0.876135 | 1.141558 | 0.999063 |
