# Supplementary material for: Identification and validation of m6A RNA methylation regulators with clinical prognostic value in Papillary thyroid cancer
Source: Cancer Cell Int. 2020 May 29;20:203. doi: 10.1186/s12935-020-01283-y (PMC7260751; doi:10.1186/s12935-020-01283-y)
Supplement: Supplementary file 10 — Additional file 10: Table S7. The multivariate Cox coefficients of MTHFD1, IGF2BP2, STT3A and GSTM4. [file 12935_2020_1283_MOESM10_ESM.docx]

**Table S7 The multivariate Cox coefficients of MTHFD1, IGF2BP2, STT3A and GSTM4.**

| Id | Coef | HR | HR.95L | HR.95H | P Value |
| --- | --- | --- | --- | --- | --- |
| MTHFD1 | 0.390258 | 1.477362 | 1.093473 | 1.996026 | 0.011021 |
| IGF2BP2 | 0.166328 | 1.180961 | 1.052696 | 1.324853 | 0.004577 |
| STT3A | -0.13296 | 0.875496 | 0.770975 | 0.994187 | 0.04038 |
| GSTM4 | 0.152319 | 1.164532 | 0.981917 | 1.381109 | 0.080071 |
